# Olive Fruit Fly (*Bactrocera oleae*) Population Dynamics in the Eastern Mediterranean: Influence of Exogenous Uncertainty on a Monophagous Frugivorous Insect

**DOI:** 10.1371/journal.pone.0127798

**Published:** 2015-05-26

**Authors:** Mariano Ordano, Izhar Engelhard, Polychronis Rempoulakis, Esther Nemny-Lavy, Moshe Blum, Sami Yasin, Itamar M. Lensky, Nikos T. Papadopoulos, David Nestel

**Affiliations:** 1 Fundación Miguel Lillo, Miguel Lillio 251, T4000JFE San Miguel de Tucumán, Tucumán, Argentina; 2 Institute of Plant Protection, ARO, The Volcani Center, Bet Dagan, Israel; 3 Department of Geography and Environment, Bar-Ilan University, Ramat Gan, Israel; 4 Agro, Nippon International Cooperation for Community Development (NICCOD), Zababdeh Office, Tubas, Palestine; 5 Laboratory of Entomology and Agricultural Zoology, University of Thessaly, Volos, Greece; 6 Consejo Nacional de Investigaciones Científicas y Técnicas, CCT Tucumán, Unidad Ejecutora Lillo (FML-CONICET), Miguel Lillo 251, T4000JFE San Miguel de Tucumán, Tucumán, Argentina; University of Crete, GREECE

## Abstract

Despite of the economic importance of the olive fly (*Bactrocera oleae*) and the large amount of biological and ecological studies on the insect, the factors driving its population dynamics (i.e., population persistence and regulation) had not been analytically investigated until the present study. Specifically, our study investigated the autoregressive process of the olive fly populations, and the joint role of intrinsic and extrinsic factors molding the population dynamics of the insect. Accounting for endogenous dynamics and the influences of exogenous factors such as olive grove temperature, the North Atlantic Oscillation and the presence of potential host fruit, we modeled olive fly populations in five locations in the Eastern Mediterranean region. Our models indicate that the rate of population change is mainly shaped by first and higher order non-monotonic, endogenous dynamics (i.e., density-dependent population feedback). The olive grove temperature was the main exogenous driver, while the North Atlantic Oscillation and fruit availability acted as significant exogenous factors in one of the five populations. Seasonal influences were also relevant for three of the populations. In spite of exogenous effects, the rate of population change was fairly stable along time. We propose that a special reproductive mechanism, such as reproductive quiescence, allows populations of monophagous fruit flies such as the olive fly to remain stable. Further, we discuss how weather factors could impinge constraints on the population dynamics at the local level. Particularly, local temperature dynamics could provide forecasting cues for management guidelines. Jointly, our results advocate for establishing monitoring programs and for a major focus of research on the relationship between life history traits and populations dynamics.

## Introduction

Despite of the economic importance of Tephritidae fruit flies (“true fruit flies”), the factors governing their population dynamics (i.e., population persistence and regulation) have seldom been subject to in-depth analytical investigation. Three main factors are expected to drive the dynamics of fruit fly populations. First, the fact that they are ectothermic organisms makes flies sensitive to climatic variation [1, 2]. Second, fruits, the “larval host”, are the initial substrate for the new generations of flies and, therefore, fruit-related variables function as demographic and selective filters in terms of natural selection. Characteristics of the host tree and fruits, for instance, affect fruit fly reproduction in numerous ways and at several levels of scale [3–6]. Third, most insect populations express what is classically termed direct density-dependence dynamics [7–10]. That is, population fluctuations depend directly on the previous population size, which is determined by intrinsic ecological processes [11]. Until now, few studies have attempted to shed light on the effects of these factors on the population dynamics of fruit flies through the use of modern analytical tools. Recently, Aluja et al. [6] published a comprehensive report on the population dynamics of three Tephritidae species of *Anastrepha* in the area of Veracruz, Mexico and related fluctuations in those populations to direct density-dependence and seasonal feedback processes. Their study demonstrated the importance of the effects of exogenous factors on the population dynamics of the three oligophagous and polyphagous species, as well as the effect of global climatic processes on the uncertainty inherent in predictions of population trends at a local and regional level. Although Aluja et al.’s study [6] contributed to our understanding of fruit fly population dynamics, the diversity of natural histories and ecologies encountered among fruit fly species, their broad geographic distribution and their economic importance have led fruit-fly ecologists to continued efforts to expand upon the current understanding of the population dynamics of this family of insects.

The present study is the first one that analyzes in deep the joint role of intrinsic and extrinsic factors on the population dynamics of the olive fly [*Bactrocera oleae* (Rossi)], a practically monophagous species of African origin. Its main host is the olive tree *Olea europaea* L., which is found throughout Africa, the Mediterranean and the Americas. Secondary hosts include some wild relatives of olive [*Olea europaea* subsp. *africana* (Mill.), *O*. *verrucosa* (Willd.)] that are found only in the fly’s area of origin [12, 13]. Uncontrolled olive fly populations may cause up to 90% damage in commercial groves [14]. This damage takes the form of fruit loss or a decrease in olive oil quality [15, 16]. A better understanding of the population dynamics of the olive fly, in terms of the joint effects of endogenous and exogenous processes, could assist in the design of management schemes for use against this pest, particularly in view of uncertain climatic scenarios and environmentally conscious markets for olive products [16, 17].

The phenology of the olive fly in the Mediterranean has been studied at several sites, including a number of sites in Greece, Italy and Spain [18–20]. The size of the olive fly population increases during the summer, after blooming and pit-hardening, and the flies thrive throughout the summer and fall [12]. In most parts of the Mediterranean region, population levels decline over the winter, with very little to no trapping of adult flies during the cold months. Substantial numbers of adults usually re-appear mid-spring [12]. Depending on temperature conditions, three to five generations per year are expected in the Mediterranean region [14]. The olive fly is sensitive to high temperatures and temperatures above 31°C induce mortality of all stages of the fly and significantly reduce its reproductive activity [21–23]. Temperatures between 25 and 29°C are optimal for reproduction, flying and development. Below 23°C, reproduction and general activity decrease sharply and these activities cease completely at 17°C [24, 25]. In olive-producing areas with elevation clines, and therefore temperature gradients, olive-fly trapping has been shown to be seasonal and linked to the optimal range of temperature, which varies with elevation and season [18, 26]. Reproductive dormancy (quiescence) has been reported in olive fly populations from Greece during periods of host unavailability, between old fruit drop and the pit-hardening of newly formed fruits [27–33]. We have also recently confirmed under field conditions the possibility of olive fly reproductive dormancy in Israel, which was expressed in field populations by a lack of male attraction to pheromone and by the absence of mature eggs in trapped females [34]. Where temperature allows, olive flies can still be trapped during the winter and spring months [35]. According to this characterization of the fly’s response to climatic variation and seasonality, olive-fly population dynamics are expected to be strongly influenced by local climatic effects and seasonal factors.

Population dynamics affected by climatic drivers can be better understood when global teleconnection climatic indicators are taken into consideration [6, 36, 37]. This approach becomes essential when global warming is predicted and weather uncertainty increases the concern on the understanding of regional weather influences on the organisms’ population dynamics [1, 37, 38]. So, together with local weather conditions, we also focused our analysis on the potential effects of the North Atlantic Oscillation (NAO), a global climatic indicator that has been shown to affect the population dynamics of *Anastrepha* fruit fly species [6] and recognized as an important factor affecting plant and animal populations in the Mediterranean region [39–41].

The Eastern Mediterranean region, specifically Israel and Palestine, includes a variety of geo-climatic regions. The region includes north-south mountain chains, a long coastline that is affected by the Mediterranean Sea and its humidity, and precipitation gradients extending from west to east and from north to south [42]. Olive trees can be found throughout a multitude of ecosystems that range from typical continental Mediterranean habitats with temperate summers and winters to desert areas with harsh summer temperatures and cold winters [16]. Elevation also creates temperature clines. This wide range of environmental conditions provides an opportunity to model and study the population dynamics of the olive fly and the importance of climatic factors.

To the best of our knowledge, no study has ever attempted to analytically characterize the autoregressive processes and population dynamics of the olive fly with respect to local climatic factors and NAO variation. Therefore, the main goals of the study were: 1) to describe the temporal profiles of olive fruit fly populations in the Eastern Mediterranean and 2) to determine the degree to which local temperature, NAO, the presence of fruit and seasonal factors drive olive-fly population dynamics, taking into account the insect’s endogenous population dynamics.

## Materials and Methods

### Olive fly sampling

**Fig 1:**
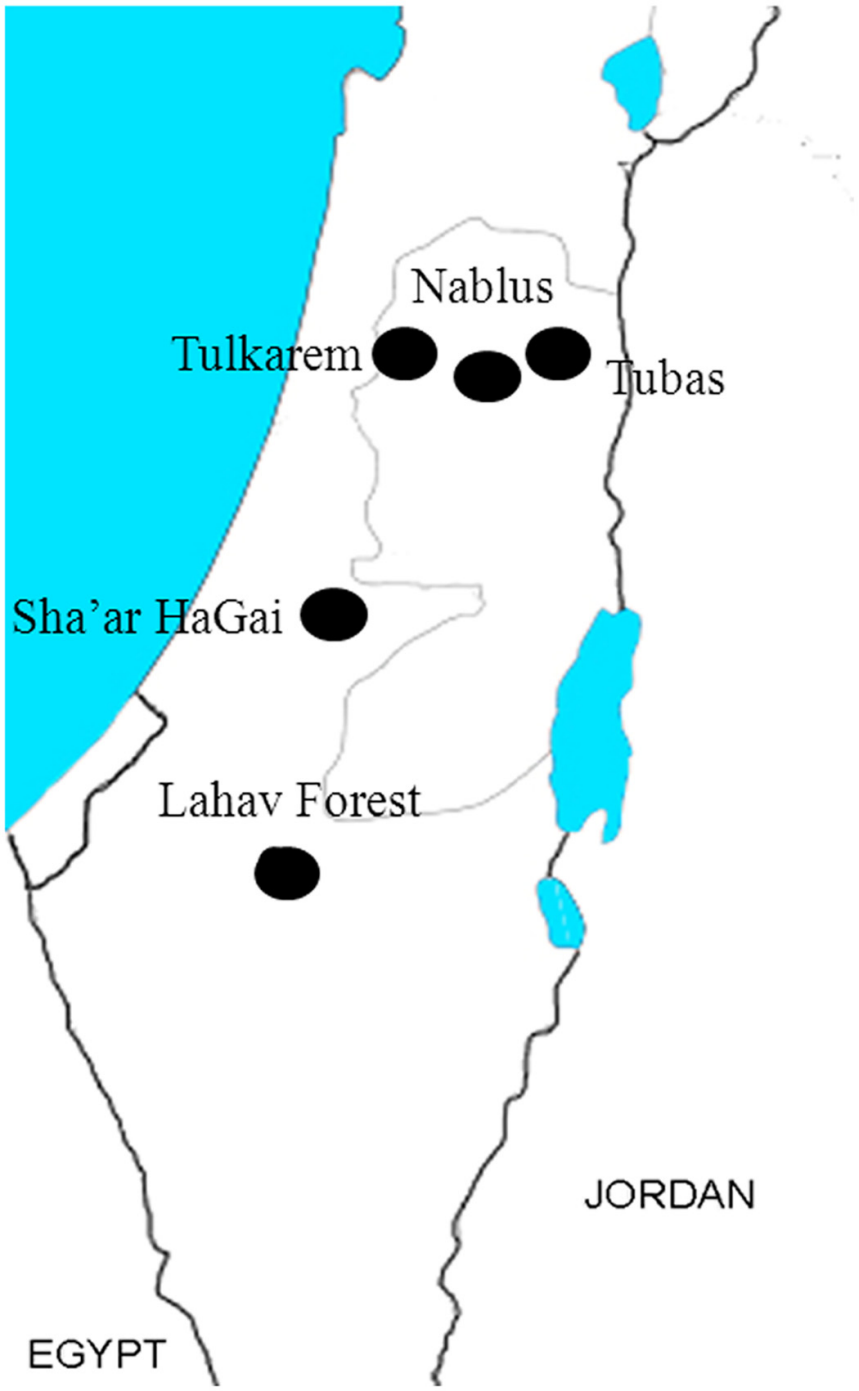
Sampling sites. Drawing of the Eastern Mediterranean (Israel, Palestine, Jordan and Egypt) showing the location of the five sampling sites.

Five locations within Israel and Palestine were selected for intensive sampling: Lahav (sampling authorized by the Forest Department, KKL), Sha’ar HaGai (sampling authorized by the Forest Department, KKL), Nablus (sampling authorized by the owner of the land), Tubas (sampling authorized by the owner of the land) and Tulkarem (sampling authorized by the owner of the land) (Fig 1). The selected locations represent different Mediterranean environments [42], spreading from west to east and from north to south (see Fig 1). Some groves contained more than one type of olive variety, but invariably one cultivar was dominant [‘Nabali-Baladi’ in the three Palestinian orchards (Nablus, Tubas and Tulkarem), ‘Manzanillo’ in Sha’ar HaGai and ‘Manzanillo’ and ‘Nabali-Muchasan’ in Lahav)]. These varieties are all considered highly susceptible to olive fly infestation [43]. The selected orchards were not treated with pesticides or irrigated.

Olive fly populations were followed by systematic sampling with yellow sticky-traps (Rimi, Israel) [44]. At each location, several sampling stations were designated for the entire study period. The locations of the sampling stations were kept constant and exposed traps were replaced approximately every 2 weeks. This was done in order to reduce sampling biases derived from changes in the attractiveness of the trap as a result of dust sticking to it. The collected traps were taken to the laboratory, where the numbers of male and female olive flies on each trap were counted.

The number of sampling stations differed between locations and was associated with the density of the trees in each grove and the size of the grove. For example, groves like the one in Sha’ar HaGai, with a high planting density (at least twice the usual, i.e., 400 trees/hectare), hosted 16 sampling stations. There were eight stations at the Lahav site and the sites in Tubas, Nablus and Tulkarem each hosted five sampling stations (in these areas, the planting density was half to one-third that used in Sha’ar HaGai). Sampling in Lahav continued over a period of 60 months, sampling in Sha’ar HaGai continued for 30 months, sampling in Tubas continued for 28 months, sampling in Nablus continued for 24 months and sampling in Tulkarem continued for 23 months. Differences in sampling times were due to logistical conditions at each location.

Sticky traps have been shown to be a rather poor method for controlling olive fly populations [45], but good for monitoring purposes because the numbers of trapped flies correlate with fruit infestation levels [46]. Unlike traps that utilize pheromone or food attractants, sticky traps are not affected by ambient conditions. Therefore, they can more accurately assess the size of adult populations throughout the year. For this reason, they are broadly used in the Eastern Mediterranean region and, based on our systematic monitoring, we assumed that the number of trapped flies is representative of the real unknown population in the grove. The number of flies caught in each trap per day (*FTD*) per location was calculated by adding the captures from all traps in each location and dividing the count by the number of traps and exposed days. Time series for each location and month were then calculated by multiplying the average *FTD* by the number of days in that month.

### Olive fruit seasonality and climatic data

We defined fruit availability in terms of the presence or absence of susceptible olive fruits in a given month (because it was not possible to acquire quantitative fruit presence data for each study site). During the study, the fruiting period was mostly stable. That is, the period between pit-hardening (which makes the fruit susceptible to oviposition) and natural fruit drop had a predictable pattern throughout the study region, with a small amount of variability of approximately 2–3 weeks due to location and variety [47]. For a local climatic indicator, we used monthly averaged air temperature. Among available meteorological data, we selected the night land surface temperature (*nLST*) described in [42], which estimates the tree canopy temperature at a resolution of 1 pixel. This temperature proxy is a more accurate temperature estimator at the grove level than data obtained from established meteorological stations [42]. The use of other local indicators, such as midday temperature, has led models to significantly inflate the variance.

As a global climatic indicator, we used the North Atlantic Oscillation index (*NAOi*), derived from data available from the U.S. National Weather Service [48]. *NAOi* is a robust pattern of recurrent atmospheric oscillations in the North Atlantic region and a good global climatic indicator used in ecological time-series [36]. In general, more rainfall and higher temperatures are associated with positive *NAOi* values [49]. Climatic effects related to *NAOi* have been reported for *Anastrepha* species [6] and populations of other species in the Mediterranean region [40, 41]. The full data set is available as S1 Table.

### Statistical analysis

To analyze olive fly population trends, we described the temporal profile of the population in each location, taking into account population fluctuations, linear trends and auto-correlation functions estimated using the rate of population change. We based the analysis on olive fly *FTD* values. The temporal profile for *FTD* was explored graphically by applying a loess function with a span of the length of the series divided by two. After describing the temporal profile with *FTD* variation, population estimates were made based on monthly numbers of captured flies per trap, which were transformed to natural log of X + 1 (hereafter *MFT*). This transformation allows time-series to be continuous in the absence of captures by transforming no-capture values to zero (actual observed occasions with no captures at all: 1 in Lahav, 1 in Sha’ar HaGai, 7 in Nablus, 2 in Tubas; S1 Table). This transformation also stabilized the variance (more information about potential biases caused by sampling errors is provided below).

The *MFT* may be considered a direct measure of the population in a given month. During the summer and fall months, the monthly frequency was also considered to be a period-lag sufficient to produce the following olive fly generation. Temporal trends were analyzed by means of simple regressions between time and the differentiated time series at lag 1 of *MFT* data (*MFT*_*t-1*_). Temporal explorations and trend analysis were also applied to climatic time-series (S1 and S2 Tables, S2 Fig). We also characterized the time series using autocorrelation functions (*ACF*) and partial rate correlation functions (*PRCF*); this was done as an initial step toward the detection of dependency between observed population dynamics and population density and seasonality [50]. For both *ACF* and *PRCF*, we used the residuals of the regression models for temporal trends, adding to each value the mean of the differentiated time series of *MFT*_*t-1*_ data. *PRCF* were constructed by replacing the parameter estimate at lag 1 from a partial autocorrelation function by the correlation between the residuals of the regression model for a temporal trend and the *MFT*_*t-1*_ data [50].

We used a general deterministic approach to investigate the influence of endogenous and exogenous influences on olive-fly population trends. Increasing or decreasing changes in the population estimates or the magnitude and sign of parameter estimates related to the rate of population change may be associated with changes in the population and/or known seasonal effects that can be modelled using general regression models [51]. We modeled the endogenous and exogenous dynamics together as general functions based on the *R*-function [11]. The *R*-function may be interpreted as the per capita growth rate or the rate of population change (*R*_*t*_) resulting from individual survival and reproductive processes [11]. We estimated *R*_*t*_ as

$$R_{t}=\;log\left(N_{t}\right)\;–\;log\;\left(N_{t-1}\right)$$ (1)

where *N*_*t*_ is the population estimate (*MFT*) and *t* is its corresponding time lag [11, 52]. Note that, in the equation above, *R*_*t*_ is equal to the differentiated time-series at lag 1 of *MFT* data (*MFT*_*t-1*_).

To evaluate the joint influences of density-dependent processes (i.e., endogenous dynamics), local and global climatic factors and seasonal effects on *R*_*t*_, we applied a generalized least squares model [50, 53–56]. This analytical tool represents a reliable alternative for studies of relatively short time-series, such as the ones presented in the current study. This may be of importance in geographic areas such as the Eastern Mediterranean, where field logistics for constant trap-monitoring may be complex. Generalized least squares allow the characterization of both the error and variance structures. These two conundrums are fundamental to dealing with errors in population estimates and the autoregressive processes that typify time-series. Without variance or correlation structure, the residuals (*ε*) are assumed to show the typical Gaussian attributes with mean zero and fixed variance unit as *ε ~ N(0*, *σ* ^*2*^ *)*. For different error variances, as expected in ecological time-series, the model allows for different variance structures [for example, *ε ~ N(0*, *σ* ^*2*^_*j*_ *)*, where *j* is each season (identity variance structure)]. Another important point is the violation of error independence given by the temporal structure of the study. Independence means that the covariance between points in time equals zero, but time-series have dependent observations. To resolve this problem and in order to model the correlation between residuals of different time points, the generalized-least-squares model may also include a correlation function (details in [53, 55]).

The model selection procedure included the following main model structure:

$$R_{t}=\;log\left(MFT_{t-1}\right)\;+\;nLST\;+\;NAOi\;+\;fruit$$ (2)

where *MFT*_*t-1*_ represents the *MFT* data corresponding to the lag 1.

We included fruit availability as the unique seasonal explanatory factor because preliminary analysis revealed a significant degree of co-linearity between this factor and the season (VIF > 18). Seasonal climatic behaviors in the grove [42], the typical fruit crop season and inter-annual variation suggest that there may be seasonal or annual effects on *R*_*t*_. Then, we incorporated a weight depicting a specific error variance structure corresponding to different error variances between seasons or years into competing models. If the residual variance was found to increase with a continuous explanatory variable, we also included them in the model as weights depicting different error variances for the corresponding continuous variable. Competing models included correlation structures with autoregressive and moving average processes (*ARMA* structure) at lag 1 or lag 2.

For model selection, we mostly followed the procedure described by Zuur et al. [55]. After each run with different correlation and/or variance structures, models were selected based on *ML* estimation and validated by residual inspection. Nonsignificant explanatory variables were rejected after a first selection of models. If two models showed no significant differences between them by means of ML, we selected the most parsimonious one [for example, an *ARMA* structure *(1*,*0)* when it did not differ from a *(1*,*1)* alternative *ARMA* structure]. We also applied ACF to residuals to inspect white noise as an aide to select the best model (white noise considered as optimal). The complete procedure was repeated to meet an optimal model, in terms of Gaussian residuals and error structure [55]. Then, the selected more-parsimonious model run by *ML* was fitted by *REML* estimation. The selected model represents the joint effects of endogenous and exogenous factors, accounting for the variation in the rate of population change over time in each location. More details on generalized-least-squares model specification and the procedure for model selection are provided in S3 Table.

Due to the fact that sampling error might lead to biases in the estimates of endogenous dynamics [57], further checks were applied to the generalized least squares models. Although the correlated structure involved in each model may reduce those biases, we corroborated this potential problem by means of a simulation-extrapolation analysis [58]. This allowed us to make inferences regarding the effects of sampling error on the estimates of endogenous effects. These analyses were run as a linear model on the main basic model (2) using *log(MFT*_*t-1*_ *)* as a simex variable, the standard deviation of monthly fly captures as an estimate of measurement error, λ = c(0.5, 1, 1.5, 2, 2.5, 3), a quadratic fit and 100 iterations. All graphics and analyses were processed in R 3.1.2 [59]. Generalized least squares were analyzed with the *gls* library of the *nlme* package [60] and simulation-extrapolation was performed with the *simex* package [61].

## Results

### Olive fly temporal patterns and trends

**Fig 2:**
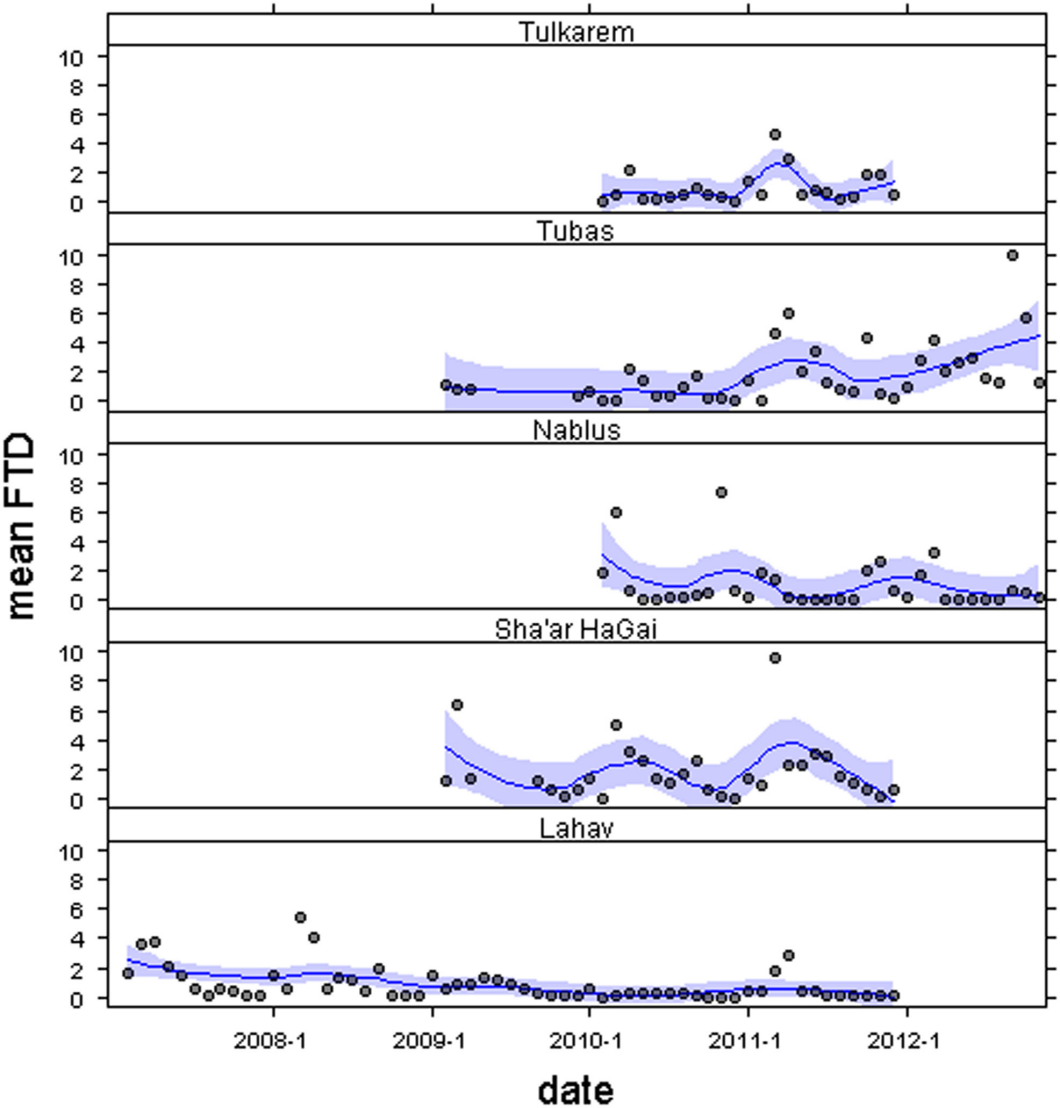
Time-series exploration. Profiles of monthly mean numbers of *Bactrocera oleae* flies/trap/day (FTD) between February 2007 and November 2012 for each site. The mean blue line has been adjusted with a loess function (weighted least square) with a 0.5 span and confidence bands with a 0.95 level of standard error. Ticks along the x-axis indicate January of the corresponding year.

**Fig 3:**
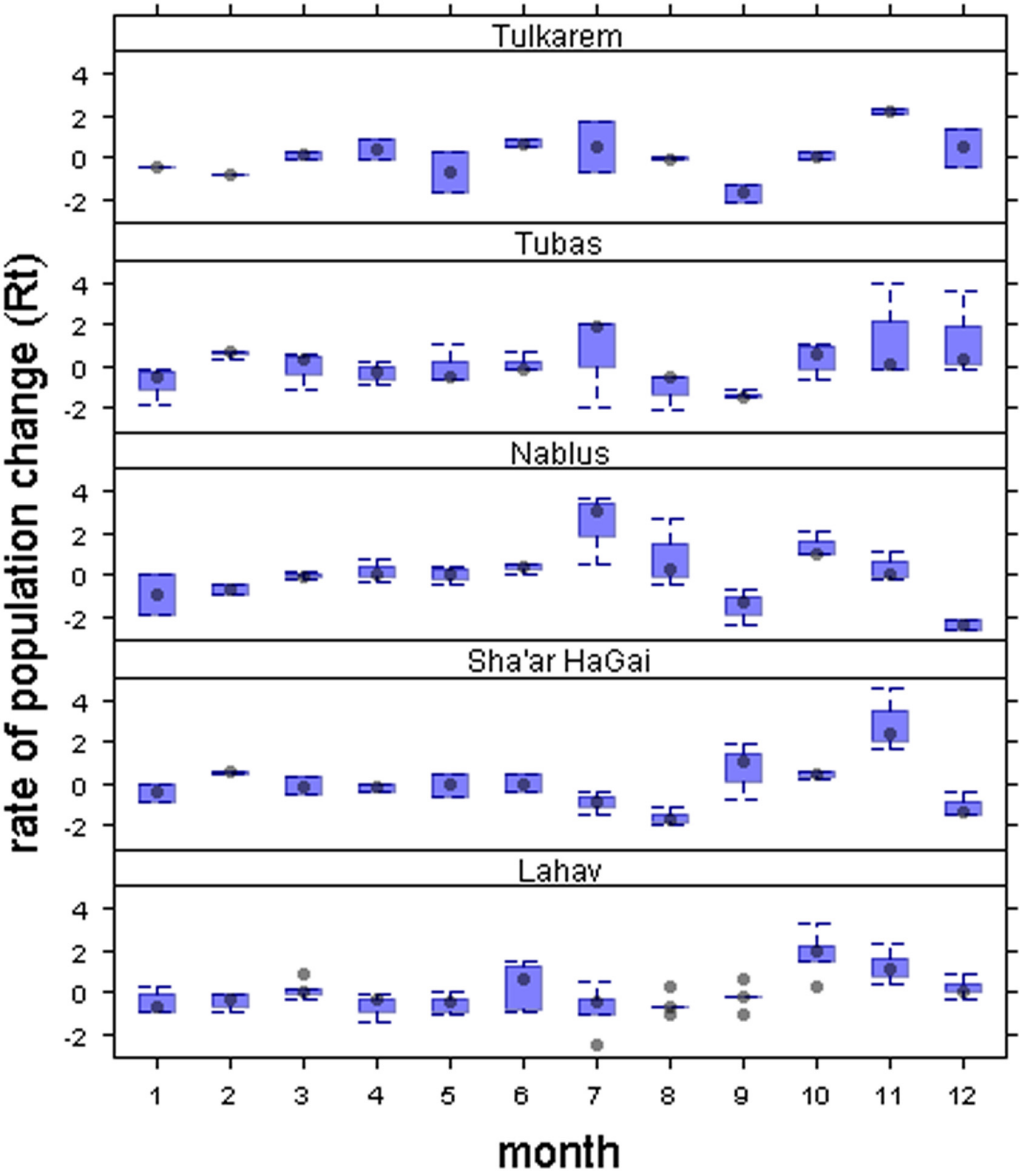
Monthly variation profiles. Monthly rate of population change (*R*t) of *Bactrocera oleae* at each site. Points represent the means, boxes represent ± 1 SE and whiskers represent ± 1 SD.

The patterns of olive fly catches were different at the five sites (Figs 2 and 3). At Lahav, Sha’ar HaGai and Tulkarem, flies were present and trapped throughout the entire study period. In contrast, in Nablus, there were five months in which no flies were captured and, in Tubas, there was one month in which no flies were caught (Fig 2). The monthly mean *FTD* values recorded at Sha’ar HaGai and Tubas were twice the values recorded at the other three sites. Descriptive statistics for each site are summarized in Table 1.

**Table 1:** Descriptive statistics for olive fly captures in the five locations in the Eastern Mediterranean region (see Fig 1).

Location	Variable	Mean	SD	Max	Time-series	Months
Lahav	Total	0.82	1.14	5.51	Feb-2007—Dec-2011	59
	females	0.33	0.48	2.15		
	males	0.46	0.65	3.20		
Sha'ar HaGai	Total	1.82	2.03	9.54	Jun-2009—Dec-2011	31
	females	0.60	0.48	1.65		
	males	1.23	1.71	8.18		
Nablus	total	0.95	1.68	7.27	Feb-2010—Nov-2012	34
	females	0.32	0.58	2.90		
	males	0.63	1.13	4.58		
Tubas	total	1.80	2.07	10.00	Sep-2009—Nov-2012	39
	females	0.61	0.70	3.62		
	males	1.19	1.42	6.39		
Tulkarem	total	0.94	1.11	4.67	Feb-2010—Dec-2011	23
	females	0.31	0.27	1.06		
	males	0.63	0.89	3.97		

Mean, standard deviation (SD) and maximum (Max) monthly average number of flies/trap/day (*FTD*) observed in each time series.

In general, in Tulkarem and Tubas, monthly mean *FTD* values increased over time, a decreasing trend was observed in Lahav and no clear trends were observed in Nablus or Sha’ar HaGai (Fig 2). These patterns, however, are apparent when we consider the linear trend analyses that indicate that there were no significant temporal patterns in *R*_*t*_ (-0.048 < adjusted *R* ^2^< -0.006; 0.002 < *F* < 0.680; 0.41 < *P* < 0.96). Broadly, the rate of population change appeared stable, but varied greatly between months (Fig 3).

**Fig 4:**
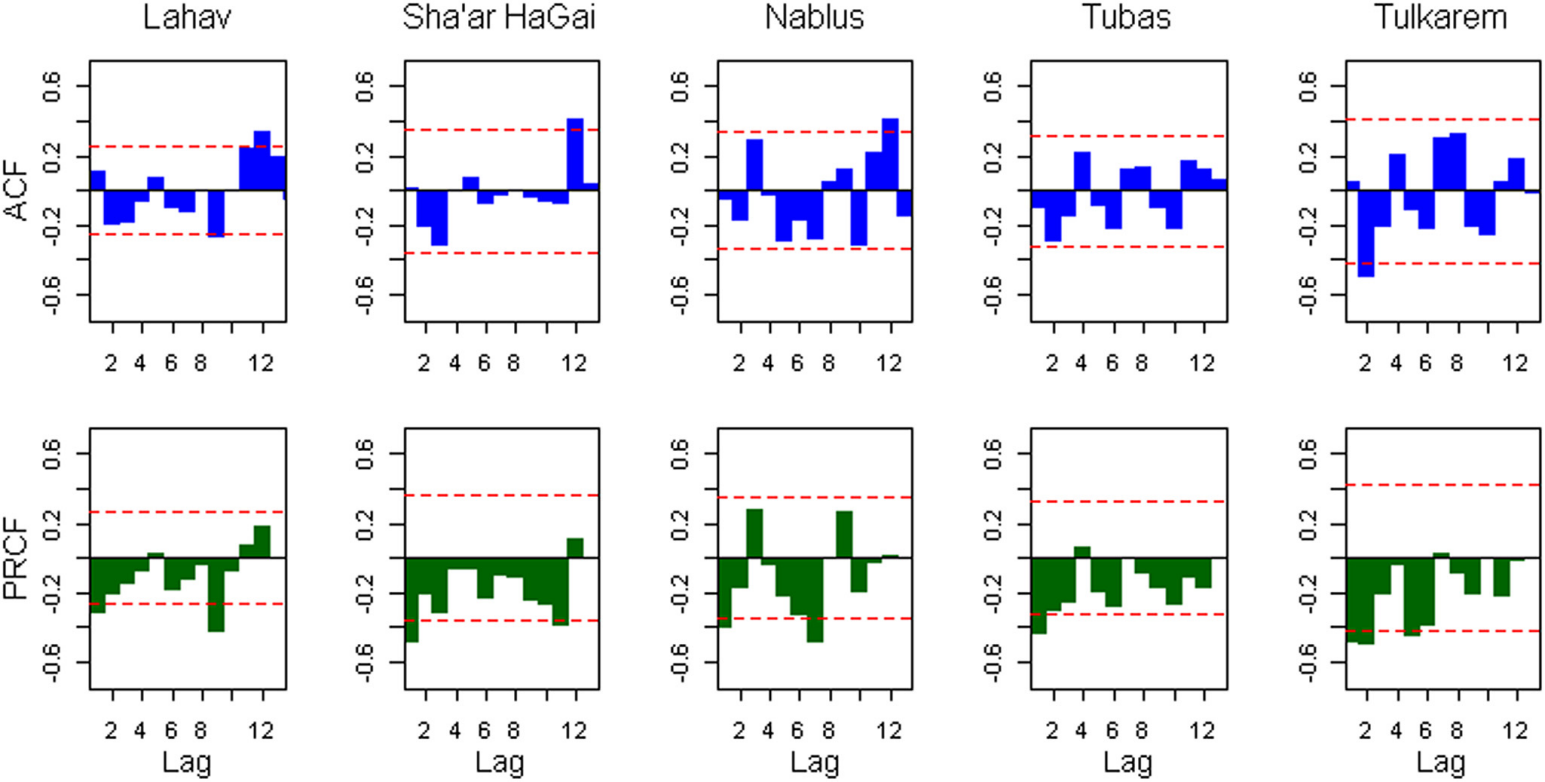
Autocorrelation functions. Autocorrelation functions (ACF, blue) and partial rate correlation functions (PRCF, green) on the de-trended rate of population change (Rt) of the olive fly in the Eastern Mediterranean region. Broken red lines depict the 95% confidence interval for the correlations.

From the ACF and PRCF profiles, it is evident that the rate of population change (*R*_*t*_) reflects different endogenous and moving average autoregressive processes between sites (Fig 4). As can be seen from the ACF profile, in Lahav, Sha’ar HaGai and Nablus, *R*_*t*_ was characterized by a positive autoregressive process on an annual scale; whereas no autoregressive effects were observed in Tubas or Tulkarem (Fig 4). However, as can be observed from the PRCF, the simplest dynamics were observed in Tubas, with a significant partial rate correlation function at lag 1 (Fig 4). Delayed autoregressive higher-order processes were also observed at the other four sites (Fig 4).

### Endogenous and exogenous factors jointly influence the rate of population change

**Fig 5:**
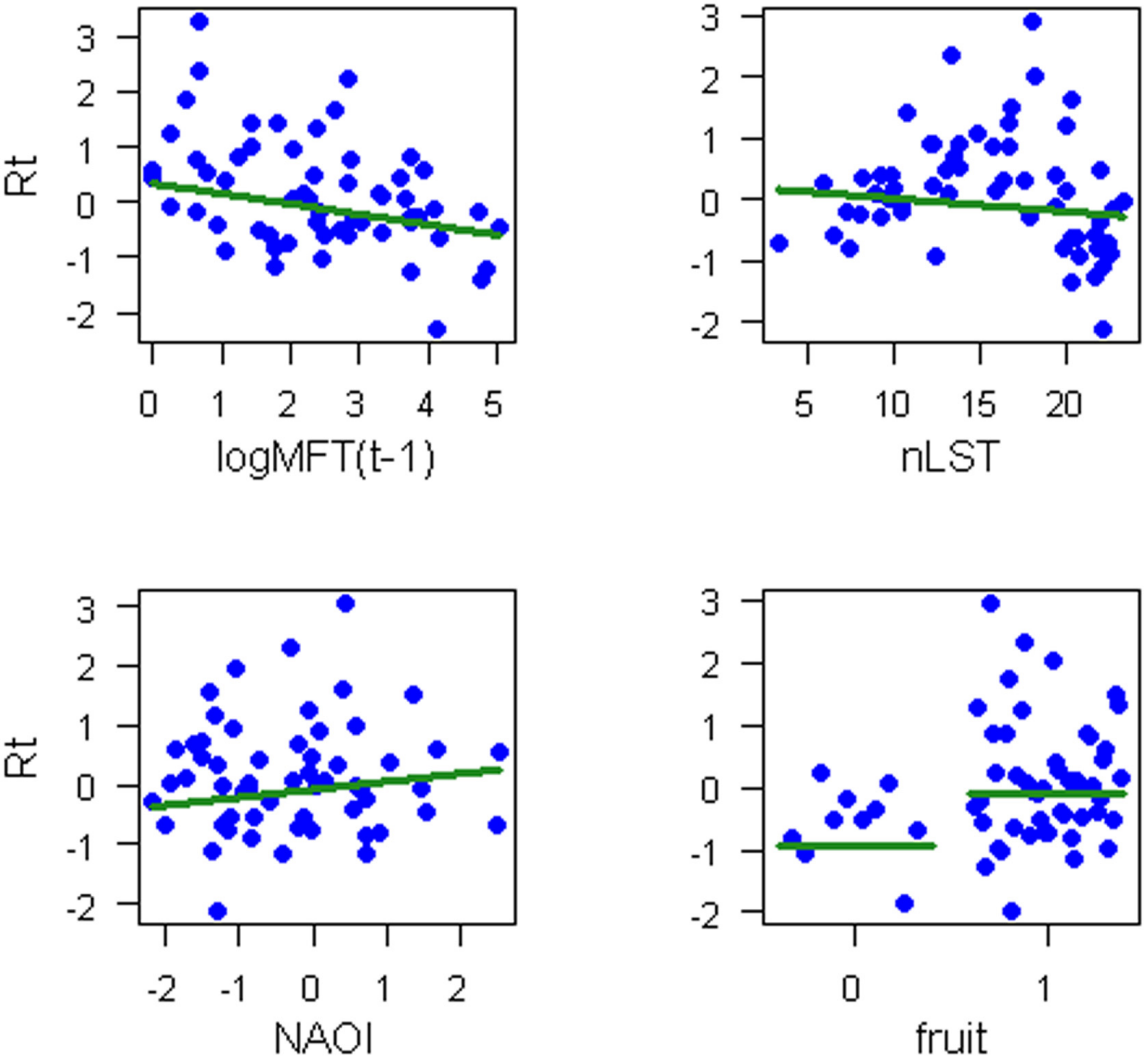
Endogenous and exogenous influences on the rate of population change. Direct endogenous (logMFTt-1) and exogenous (nLST; NAOi; fruit availability, 0: absent, 1: present) influences on the rate of change in the olive fly population (*R*t) in Lahav. Observed points are depicted in blue. The fitted line is drawn in green, according to the selected generalized-least-squares model indicating partial regressions (Table 2).

The generalized-least-squares model fitting showed that direct first-order endogenous dynamics were present at all five locations and that climatic factors significantly contribute to olive fly population dynamics (Table 2). In addition, underlying endogenous dynamics of a higher order are suggested by the error-correlated *ARMA* structures of the optimal resulting models for Lahav and Sha’ar HaGai. With the exception of Tulkarem, *nLST* appeared to be a significant driver of olive fly dynamics. The effect of *nLST* was negative in Lahav and Sha’ar HaGai, but positive in Nablus (Table 2), a difference probably related to geography. (Nablus is located at a higher altitude than the other locations). Exogenous direct influences of *NAOi* and fruit availability on olive-fly population dynamics were only observed only at Lahav, where they were positive (Table 2, Fig 5). Details regarding the specification of the generalized-least-squares model and model selection are provided in S3 Table.

**Table 2:** Summary of selected generalized-least-squares models of the rate of change (*R*_*t*_) in *Bactrocera oleae* populations in five locations in the Eastern Mediterranean region.

Model structure		Lahav		Sha'ar HaGai		Nablus		Tubas		Tulkarem	
		*Coef (SE)*	*P*	*Coef (SE)*	*p*	*Coef (SE)*	*P*	*Coef (SE)*	*P*	*Coef (SE)*	*p*
Variables*	*log(MFT*_*t-1*_ *)*	**-0.187** (0.008)	**<0.0001**	**-0.767** (0.135)	**<0.0001**	**-0.331** (0.085)	**0.001**	**-0.975** (0.172)	**<0.0001**	**-0.825** (0.247)	**0.0039**
	Asymptotic	**-0.636** (0.167)	**0.0004**	**-1.282** (0.420)	**0.0054**	**-0.537** (0.173)	**0.0045**	**-0.628** (0.278)	**0.0308**	**-0.673** (0.296)	**0.0365**
	Jackknife	**-0.636** (0.149)	**<0.0001**	**-1.282** (0.222)	**<0.0001**	**-0.537** (0.157)	**0.0020**	**-0.628** (0.191)	**0.0025**	**-0.673** (0.298)	**0.0373**
	*nLST*	**-0.022** (0.004)	**<0.0001**	**-0.075** (0.018)	**0.0003**	**0.124** (0.019)	**<0.0001**	-0.005 (0.037)	0.8832	-0.038 (0.044)	0.3898
	*NAOi*	**0.134** (0.016)	**<0.0001**	0.067 (0.044)	0.1401	0.032 (0.097)	0.748	0.053 (0.155)	0.7329	0.172 (0.191)	0.3811
	*Fruit*	**0.833** (0.153)	**<0.0001**	-0.377 (0.234)	0.1194	0.690 (0.434)	0.124	0.185 (0.317)	0.5639	0.576 (0.596)	0.3474
ARMA structure											
*φ*_*1*_		0.889		-0.113		-0.395		0.542		0.472	
*φ*_*2*_		-0.701		-0.320		-0.394				-0.532	
*θ*_*1*_		-1.547		0.390							
*θ*_*2*_		0.547		-0.610							
Variance structure		Combination of fixed variance for *nLST* and different variance per stratum for season and year		Combination of fixed variance for *nLST* and different variance per stratum for season		Fixed variance for *Nlst*		Different variance per stratum for season		Fixed variance for *nLST*	

Each model represents a best representation of direct endogenous and exogenous influences on olive fly population dynamics. Significant coefficients (*coef*) are indicated in bold; standard errors are shown in parentheses (*SE*). The *φ*_*i*_ and *θ*_*i*_ values represent the autoregressive (AR) and moving-average (MA) parameters of the ARMA structure, respectively. See S3 Table for details. The estimates of endogenous effects derived from simulation-extrapolation analyses are shown here as the asymptotic variance and jackknife variance. See Methods for details.

* Variables: *log(MFT*_*t-1*_ *)*, mean flies per trap at lag t-1; *nLST*, night land surface temperature; *NAOi*, North Atlantic Oscillation index; *fruit*, availability of susceptible fruit.

Simulation-extrapolation analyses showed that the unbiased estimates of endogenous effects differed slightly from the generalized-least-squares estimates (Table 2). Endogenous effects were underestimated for Lahav, Sha’ar HaGai and Nablus populations and were overestimated for the Tubas and Tulkarem populations (Table 2). This suggests that measurement error might show a non-linear structure and that the rate of population change might be less stable than had been estimated.

## Discussion

Olive fly population dynamics in the Eastern Mediterranean region are characterized by population processes of the first and higher orders. Local climatic variation, measured as night land surface temperature (*nLST*), was the main exogenous driver in all populations. The influence of NAO on local population dynamics was found potentially relevant for one of the surveyed populations. In that same population, fruit availability was a significant driver of olive-fly population dynamics. For practical pest-management proposes, our results indicate recurrent olive fly infestations in spite of the entangled influences of exogenous factors. Following, we explain how these influences could be disclosed in order to understand how underlying life history characteristics could arise in each component of the complex nature of population dynamics, and to give predictable expectations for management guidelines.

Firstly, the absence of linear temporal trends in the five study locations indicates that olive fly populations in the Eastern Mediterranean are fairly stable. This stability is probably related to the close association of this monophagous species with its host, as well as the fact that these flies are able to lay eggs throughout most of the year (except for a few months in the spring, when no host is available). In addition, the ability of the fly to halt reproduction under adverse climatic and host conditions [12], especially in the spring, may allow the annual renewal of the population and enhance its stability. Moreover, spring reproductive dormancy [12, 27, 29, 34] allows the synchronization of the first and subsequent generations, a situation which probably strengthens the seasonal variation in the rate of population change in three of the five studied locations.

Similar to the dynamics observed in *Anastrepha* species [6] and the observed long-term population stability of the olive fly, linear and non-linear endogenous and exogenous influences appear to shape the fluctuations in the fly population in the Eastern Mediterranean region. All populations exhibited negative density-dependence of the first order, indicating a simple short-term response directly related to the level of reproduction in the previous month.

As expected, exogenous factors also shaped population dynamics. In general, population dynamics corresponded to the climatic patterns observed in the specific sampling locations (for more information, consult Blum et al. [42]). Our models revealed that population oscillations are mainly driven by the local temperature conditions and seasonality. Geographic location and climate are expected to have an important impact upon the ability of the population to reproduce and thrive [26]. This fact might explain the differential influence of *nLST* in the different locations. Although it is well established that temperature affects the reproduction, mortality and flying activity of the olive fly [21, 22, 24], few studies [17, 26] have in fact described the effect of different temperature regimes and environments on populations of the olive fly simultaneously developing in distinctive geo-climatic regions. Moreover, none of these studies [17, 26] analyzed the population fluctuations of the olive fly using an analytical population dynamics’ approach.

In cold areas with milder summer temperatures and cold winters and nights, such as are found at high elevations or the northern latitudes of the Mediterranean region, olive fly populations are expected to fluctuate and to be highly seasonal. That is, under these meteorological conditions, olive fly populations are expected to develop throughout the summer and autumn, but exhibit a slower rate of development, or even quit egg-laying and larval development, during the winter. This may have been the case in the grove that we studied in Nablus, in which the rate of population change was positively affected by temperature. At this high-altitude location, fly-trapping increased during the summer and fall, but almost no flies were caught during the winter and spring.

In contrast, in many valleys, lowlands, and coastline areas of the Mediterranean region, summer temperatures may be harsh, but winter temperatures fairly mild. As a result, a contrasting trapping and population development trend may be observed between seasons, with higher reproductive and flying activity during winter, spring and autumn, but reduced or no activity during the summer months. Such trends were observed at both Sha’ar HaGai and Lahav (on the border of the Negev desert), with significant seasonality in the dynamics of the population and negative linear influences of *nLST* on the rate of population change.

The results from Tubas, which lies on the eastern slopes of the West Bank, are particularly interesting. The climate in this location is mild throughout the year [42], allowing for constant activity and trapping of the population; similar to the olive fly trends reported by Burrack et al. [17] in some areas of California. This activity was reflected in our analysis as simple endogenous population dynamics when seasonal variation was taken into account.

Based on the climatic similarities between Tulkarem and Sha’ar HaGai, we expected to find similar population dynamic patterns in these two locations. Differences observed between the two sites may be related to differences in time-series lengths and differences in endogenous dynamics, which are indicated by the magnitude of the autoregressive parameters. In this sense, endogenous non-monotonic effects on population dynamics require greater attention since they may be intrinsically fueled by non-linear reproductive processes. For example, it is known that the olive fly usually lays one egg per host fruit, but under high-population pressure more than one egg may be laid per fruit [12]. This may lead to non-monotonic responses in the rate of population change driven by synergistic effects of endogenous and exogenous factors.

During the period of the study, southern Israel, including the Lahav sampling site, was affected by a drought [62]. More northern areas were less affected by drought during the period of the study. This phenomenon was probably highlighted by the *ARMA* fitted model for Lahav, which suggested that both *NAOi* and host fruit occurrence significantly affected the olive fly population dynamics at this site, but not at the other more northern study sites. It is commonly agreed that positive *NAOi* values are usually related to increasing storm activity, but positive *NAOi* is also known to strongly increase local climatic variability (i.e., higher local uncertainty), leading to sharp climatic differences between geographic areas that are close to one another [6, 63]. Thus, it is possible that while some relatively close areas in the Mediterranean region were probably suffering from heavy storms, others were affected by drought during this positive *NAOi* oscillation cycle. This might have had different effects upon the ecosystems and population dynamics of many organisms, including the olive fly [39–41]. The positive *NAOi* may have differentially affected the olive fly dynamics in the region, producing different dynamics in northern and southern olive fly populations. Drought may also explain the significant effect of host availability in Lahav’s olive fly population trends (e.g., being a rain-fed grove, olive fruit production was significantly reduced). This possibility requires further investigation. Although we did not detected NAO effects in the other localities, their synergistic effects on local population dynamics deserves a deeper investigation in order to get a broader picture for forecasting purposes.

Monophagy in fruit flies is an uncommon condition. Most of the Tephritidae fruit flies that have been studied develop in more than one host. Oligophagy and/or polyphagy increase the likelihood that fruit fly populations will overcome host shortages or other fluctuating exogenous influences, to keep their population dynamics stable over time [6, 64]. The observed results with the monophagous olive fly also suggest stable population dynamics in the Eastern Mediterranean region, despite the fact that the olive fly is dependent on the phenology of its single host. Although host fruit were available throughout most of the year, this host availability did not significantly drive the population dynamics of the olive fly in our study, with the exception of the Lahav site, where host availability was negatively affected by drought. Overcoming the short period of olive-fruit unavailability and allowing stable population fluctuations over time may require the existence of special life-history traits that compensate for host absence. Monophagy in fruit flies seems to be commonly accompanied by special life-history conditions, such as diapause (e.g., in *Rhagoletis cerasi*) [65] or other strategies that allow them to deal with periods during which host fruit are not available. In the olive fly, reproductive dormancy during spring [12, 27, 29, 34] seems to have evolved as a way to compensate for the unavailability of fruit between budding and pit-hardening. The existence of this mechanism is probably responsible for the ability of the fly population to remain stable throughout the year and to avoid collapse during periods during which no fruit are available. The characteristics of the population dynamics of the olive fly, which originated in Central Africa and then spread to more temperate regions of the world [12, 13] at other latitudes, and the way in which reproductive dormancy, which seems to be a facultative trait, is expressed in other parts of the Mediterranean region, Africa and California merit further study. Lastly, olive fly population forecasting for management purposes can be enriched by taking greater advantage of modern analytical techniques on weather estimation [42], and by establishing monitoring programs in accordance with the dynamics of life history traits.

## Supporting Information

S1 Fig Temporal explorations applied to climatic time-series. Profiles of mean night land surface temperatures (mean nLST) between February 2007 and November 2012 at each site. The red line has been adjusted with a loess function (weighted least square) with a 0.2 span and the pink confidence bands with a 0.95 level of standard error. Ticks along the date axis indicate January of the corresponding year.
(TIF)

S2 Fig Temporal explorations applied to NAOi time-series. Profile of the North Atlantic Oscillation index (NAOi) between February 2007 and November 2012. The red line has been adjusted with a loess function (weighted least square) with a 0.2 span and the pink confidence bands with a 0.95 level of standard error. Ticks along the date axis indicate January of the corresponding year.
(TIF)

S1 Table Time-series data for *Bactrocera oleae*. Datasheet with time-series data for *Bactrocera oleae* captures in five locations (Site) in the Eastern Mediterranean region. Monthly populations were estimated as the product of FTD (flies/trap/day) and days (days corresponding to each month). Fruit: the presence (1) or absence (0) of olive fruits in the orchards. nLST: average night land surface temperature. NAOi: North Atlantic Oscillation index. SH: Sha’ar HaGai location. NA: without data. See Methods section for details.
(DOC)

S2 Table Temporal explorations and trend analysis applied to climatic time-series. Linear trend analysis for monthly average night land surface temperatures between February 2007 and November 2012 at five locations in the Eastern Mediterranean region. The linear trend analyses indicated that there were no temporal trends in the monthly North Atlantic Oscillation index (adjusted *R* ^2^ = -0.01468, *t* = -0.043, *p* = 0.965) and the monthly-average night land surface temperatures. For data sources, see Methods.
(DOC)

S3 Table Summary of the procedure for the selection of the best generalized-least-squares model. Models carried out to examine the influence of denso-dependence, climatic (local and global) factors, fruit occurrence and seasonality on the rate of change in *Bactrocera oleae* populations in five locations in the Eastern Mediterranean region. Description of the procedure used to select the optimal model. Tables of competing models for each site are showed. Selected models are marked with an asterisk. Main basic model (Eq 2 of Methods): Rt = log(MFTt-1) + nLST + NAOi + fruit. BIC: Bayesian Information Criteria. ARMA: order of the autoregressive moving average (p, q). The variance structure follows the R code used for generalized-least-squares models.
(DOC)
