# Supplementary figures and images for: Olive Fruit Fly (Bactrocera oleae) Population Dynamics in the Eastern Mediterranean: Influence of Exogenous Uncertainty on a Monophagous Frugivorous Insect
Source: PLoS One. 2015 May 26;10(5):e0127798. doi: 10.1371/journal.pone.0127798 (PMC4444365; doi:10.1371/journal.pone.0127798)

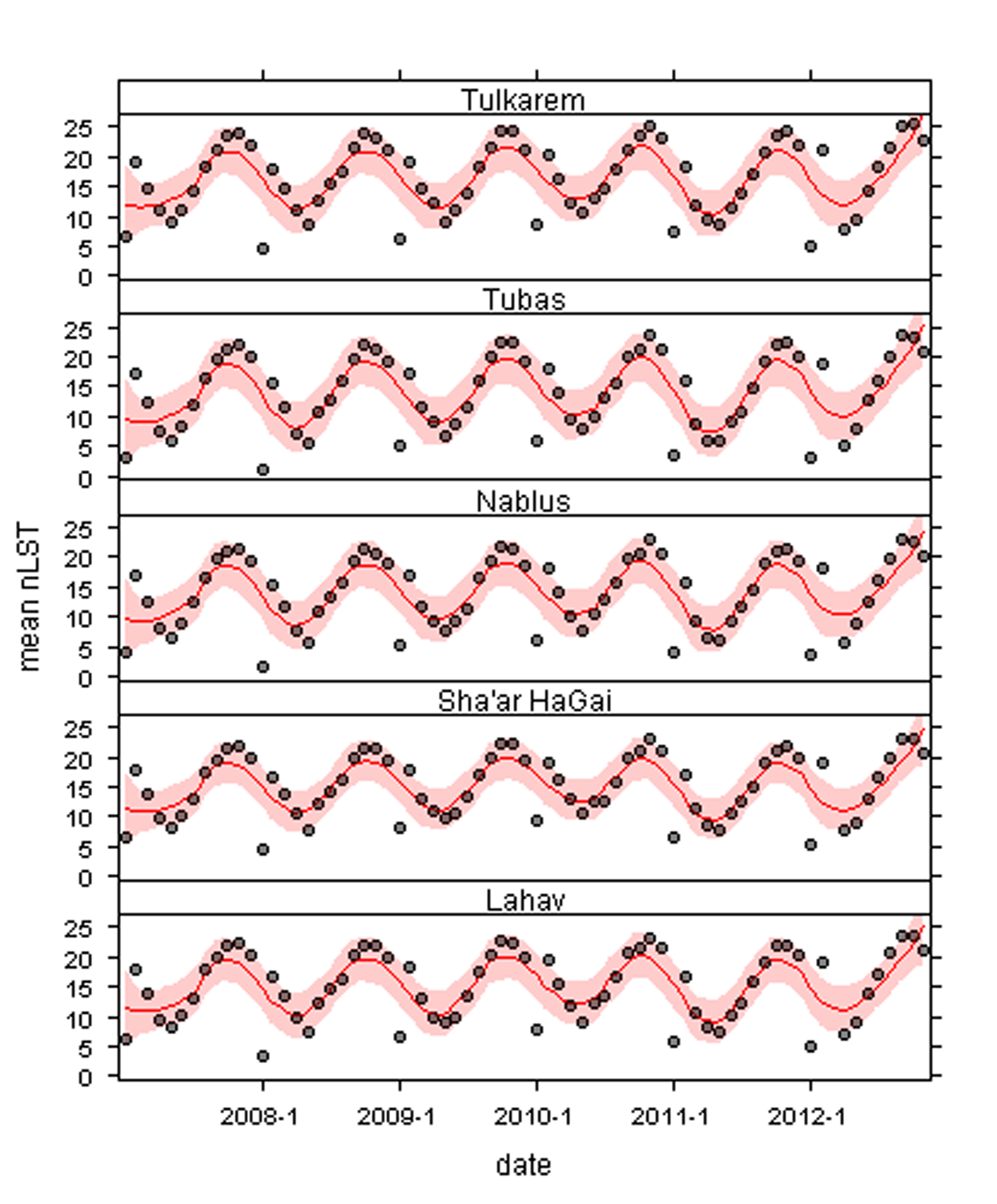

Supplement: S1 Fig — Profiles of mean night land surface temperatures (mean nLST) between February 2007 and November 2012 at each site. The red line has been adjusted with a loess function (weighted least square) with a 0.2 span and the pink confidence bands with a 0.95 level of standard error. Ticks along the date axis indicate January of the corresponding year. (TIF) [file pone.0127798.s001.tif]

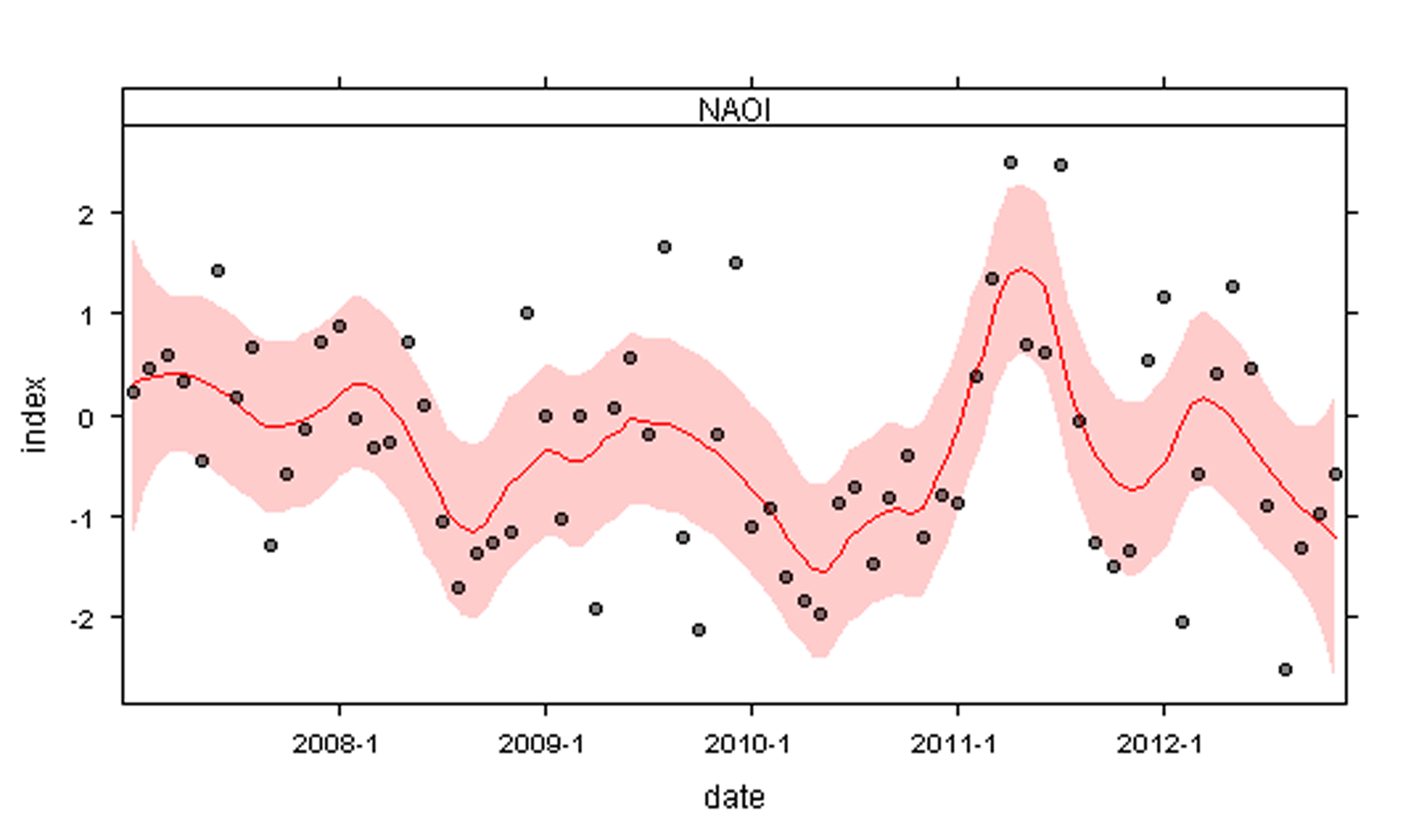

Supplement: S2 Fig — Profile of the North Atlantic Oscillation index (NAOi) between February 2007 and November 2012. The red line has been adjusted with a loess function (weighted least square) with a 0.2 span and the pink confidence bands with a 0.95 level of standard error. Ticks along the date axis indicate January of the corresponding year. (TIF) [file pone.0127798.s002.tif]
