# Supplementary material for: Olive Fruit Fly (Bactrocera oleae) Population Dynamics in the Eastern Mediterranean: Influence of Exogenous Uncertainty on a Monophagous Frugivorous Insect
Source: PLoS One. 2015 May 26;10(5):e0127798. doi: 10.1371/journal.pone.0127798 (PMC4444365; doi:10.1371/journal.pone.0127798)
Supplement: S1 Table — Datasheet with time-series data for Bactrocera oleae captures in five locations (Site) in the Eastern Mediterranean region. Monthly populations were estimated as the product of FTD (flies/trap/day) and days (days corresponding to each month). Fruit: the presence (1) or absence (0) of olive fruits in the orchards. nLST: average night land surface temperature. NAOi: North Atlantic Oscillation index. SH: Sha’ar HaGai location. NA: without data. See Methods section for details. (DOC) [file pone.0127798.s003.doc]

**S1 Table**

Title

*Olive fruit fly (Bactrocera oleae) population dynamics in the Eastern Mediterranean: Influence of exogenous uncertainty on a monophagous frugivorous insect*

Authors

Mariano Ordano, Izhar Engelhard, Polychronis Rempoulakis, Esther Nemny-Lavy, Moshe Blum, Sami Yasin, Itamar Lensky, Nikos T. Papadopoulos, David Nestel*

* Corresponding author

Datasheet with time-series data of *Bactrocera oleae* captures corresponding to five locations (Site) of the Eastern Mediterranean. Monthly populations were estimated as the product of FTD (flies/trap/day) by Days (days corresponding to each month). “Fruit” indicates the presence (1) or absence (0) of olive fruits in the orchards. nLST: average night land surface temperature. NAOi: North Atlantic Oscillation index. SH: Sha’ar HaGai location. NA: without data. See Materials and Methods section for details.

| Date | Site | FTD | Days | Season | Fruit | NAOi | nLST |
| --- | --- | --- | --- | --- | --- | --- | --- |
| Jan-07 | Lahav | NA | 31 | winter | 1 | 0.22 | 6.20 |
| Feb-07 | Lahav | 2.05 | 28 | winter | 1 | -0.47 | 8.43 |
| Mar-07 | Lahav | 1.53 | 31 | winter | 1 | 1.44 | 10.45 |
| Apr-07 | Lahav | 0.56 | 30 | spring | 0 | 0.17 | 13.08 |
| May-07 | Lahav | 0.17 | 31 | spring | 0 | 0.66 | 17.77 |
| Jun-07 | Lahav | 0.63 | 30 | spring | 1 | -1.31 | 19.93 |
| Jul-07 | Lahav | 0.36 | 31 | summer | 1 | -0.58 | 21.95 |
| Aug-07 | Lahav | 0.16 | 31 | summer | 1 | -0.14 | 22.28 |
| Sep-07 | Lahav | 0.03 | 30 | summer | 1 | 0.72 | 20.24 |
| Oct-07 | Lahav | 1.70 | 31 | autumn | 1 | 0.45 | 18.03 |
| Nov-07 | Lahav | 3.66 | 29 | autumn | 1 | 0.58 | 13.76 |
| Dec-07 | Lahav | 3.83 | 31 | autumn | 1 | 0.34 | 9.26 |
| Jan-08 | Lahav | 1.42 | 30 | winter | 1 | 0.89 | 3.25 |
| Feb-08 | Lahav | 0.54 | 29 | winter | 1 | 0.73 | 7.33 |
| Mar-08 | Lahav | 1.35 | 31 | winter | 1 | 0.08 | 12.25 |
| Apr-08 | Lahav | 1.22 | 30 | spring | 0 | -1.07 | 14.74 |
| May-08 | Lahav | 0.44 | 31 | spring | 0 | -1.73 | 16.29 |
| Jun-08 | Lahav | 1.97 | 30 | spring | 1 | -1.39 | 20.22 |
| Jul-08 | Lahav | 0.12 | 31 | summer | 1 | -1.27 | 22.06 |
| Aug-08 | Lahav | 0.04 | 31 | summer | 1 | -1.16 | 21.77 |
| Sep-08 | Lahav | 0.10 | 30 | summer | 1 | 1.02 | 19.89 |
| Oct-08 | Lahav | 0.53 | 31 | autumn | 1 | -0.04 | 16.60 |
| Nov-08 | Lahav | 5.51 | 30 | autumn | 1 | -0.32 | 13.34 |
| Dec-08 | Lahav | 4.02 | 28 | autumn | 1 | -0.28 | 9.99 |
| Jan-09 | Lahav | 1.47 | 31 | winter | 1 | -0.01 | 6.56 |
| Feb-09 | Lahav | 1.33 | 28 | winter | 1 | 0.06 | 8.86 |
| Mar-09 | Lahav | 1.19 | 31 | winter | 1 | 0.57 | 9.66 |
| Apr-09 | Lahav | 0.84 | 30 | spring | 0 | -0.20 | 13.37 |
| May-09 | Lahav | 0.53 | 31 | spring | 0 | 1.68 | 17.53 |
| Jun-09 | Lahav | 0.21 | 30 | spring | 1 | -1.21 | 20.48 |
| Jul-09 | Lahav | 0.14 | 31 | summer | 1 | -2.15 | 22.63 |
| Aug-09 | Lahav | 0.05 | 31 | summer | 1 | -0.19 | 22.46 |
| Sep-09 | Lahav | 0.03 | 30 | summer | 1 | 1.51 | 19.79 |
| Oct-09 | Lahav | 0.54 | 31 | autumn | 1 | -1.03 | 18.11 |
| Nov-09 | Lahav | 0.85 | 30 | autumn | 1 | -0.02 | 12.95 |
| Dec-09 | Lahav | 0.86 | 31 | autumn | 1 | -1.93 | 9.83 |
| Jan-10 | Lahav | 0.50 | 31 | winter | 1 | -1.11 | 7.98 |
| Feb-10 | Lahav | 0.26 | 28 | winter | 1 | -1.98 | 9.24 |
| Mar-10 | Lahav | 0.31 | 31 | winter | 1 | -0.88 | 12.30 |
| Apr-10 | Lahav | 0.21 | 30 | spring | 0 | -0.72 | 13.67 |
| May-10 | Lahav | 0.20 | 31 | spring | 0 | -1.49 | 16.60 |
| Jun-10 | Lahav | 0.06 | 30 | spring | 1 | -0.82 | 20.61 |
| Jul-10 | Lahav | 0.00 | 30 | summer | 1 | -0.42 | 21.54 |
| Aug-10 | Lahav | 0.01 | 31 | summer | 1 | -1.22 | 23.30 |
| Sep-10 | Lahav | 0.00 | 30 | summer | 1 | -0.79 | 21.55 |
| Oct-10 | Lahav | 0.01 | 29 | autumn | 1 | -0.93 | 19.38 |
| Nov-10 | Lahav | 0.10 | 28 | autumn | 1 | -1.62 | 15.65 |
| Dec-10 | Lahav | 0.28 | 31 | autumn | 1 | -1.85 | 12.06 |
| Jan-11 | Lahav | 0.35 | 31 | winter | 1 | -0.88 | 5.85 |
| Feb-11 | Lahav | 0.33 | 28 | winter | 1 | 0.70 | 7.27 |
| Mar-11 | Lahav | 0.35 | 31 | winter | 1 | 0.61 | 10.44 |
| Apr-11 | Lahav | 0.06 | 30 | spring | 0 | 2.48 | 12.31 |
| May-11 | Lahav | 0.03 | 31 | spring | 0 | -0.06 | 15.88 |
| Jun-11 | Lahav | 0.08 | 30 | spring | 1 | -1.28 | 19.31 |
| Jul-11 | Lahav | 0.16 | 31 | summer | 1 | -1.51 | 21.87 |
| Aug-11 | Lahav | 0.03 | 31 | summer | 1 | -1.35 | 21.98 |
| Sep-11 | Lahav | 0.02 | 30 | summer | 1 | 0.54 | 20.18 |
| Oct-11 | Lahav | 0.33 | 31 | autumn | 1 | 0.39 | 16.79 |
| Nov-11 | Lahav | 1.85 | 30 | autumn | 1 | 1.36 | 10.78 |
| Dec-11 | Lahav | 2.78 | 31 | autumn | 1 | 2.52 | 8.08 |
| Jan-12 | Lahav | NA | 30 | winter | 1 | 1.17 | 4.88 |
| Feb-12 | Lahav | NA | 28 | winter | 1 | 0.42 | 7.21 |
| Mar-12 | Lahav | NA | 31 | winter | 1 | 1.27 | 9.09 |
| Apr-12 | Lahav | NA | 30 | spring | 0 | 0.47 | 13.79 |
| May-12 | Lahav | NA | 31 | spring | 0 | -0.91 | 17.11 |
| Jun-12 | Lahav | NA | 30 | spring | 1 | -2.53 | 20.54 |
| Jul-12 | Lahav | NA | 31 | summer | 1 | -1.32 | 23.46 |
| Aug-12 | Lahav | NA | 31 | summer | 1 | -0.98 | 23.45 |
| Sep-12 | Lahav | NA | 30 | summer | 1 | -0.59 | 20.97 |
| Oct-12 | Lahav | NA | 27 | autumn | 1 | -2.06 | 18.92 |
| Nov-12 | Lahav | NA | 0 | autumn | 1 | -0.58 | NA |
| Jan-07 | SH | NA | 31 | winter | 1 | 0.22 | 6.44 |
| Feb-07 | SH | NA | 28 | winter | 1 | -0.47 | 8.19 |
| Mar-07 | SH | NA | 31 | winter | 1 | 1.44 | 10.14 |
| Apr-07 | SH | NA | 30 | spring | 0 | 0.17 | 12.83 |
| May-07 | SH | NA | 31 | spring | 0 | 0.66 | 17.36 |
| Jun-07 | SH | NA | 30 | spring | 1 | -1.31 | 19.44 |
| Jul-07 | SH | NA | 31 | summer | 1 | -0.58 | 21.22 |
| Aug-07 | SH | NA | 31 | summer | 1 | -0.14 | 21.75 |
| Sep-07 | SH | NA | 30 | summer | 1 | 0.72 | 19.88 |
| Oct-07 | SH | NA | 31 | autumn | 1 | 0.45 | 17.65 |
| Nov-07 | SH | NA | 29 | autumn | 1 | 0.58 | 13.84 |
| Dec-07 | SH | NA | 31 | autumn | 1 | 0.34 | 9.86 |
| Jan-08 | SH | NA | 30 | winter | 1 | 0.89 | 4.34 |
| Feb-08 | SH | NA | 29 | winter | 1 | 0.73 | 7.77 |
| Mar-08 | SH | NA | 31 | winter | 1 | 0.08 | 12.15 |
| Apr-08 | SH | NA | 30 | spring | 0 | -1.07 | 14.36 |
| May-08 | SH | NA | 31 | spring | 0 | -1.73 | 16.16 |
| Jun-08 | SH | NA | 30 | spring | 1 | -1.39 | 19.62 |
| Jul-08 | SH | NA | 31 | summer | 1 | -1.27 | 21.59 |
| Aug-08 | SH | NA | 31 | summer | 1 | -1.16 | 21.36 |
| Sep-08 | SH | NA | 30 | summer | 1 | 1.02 | 19.28 |
| Oct-08 | SH | NA | 31 | autumn | 1 | -0.04 | 16.71 |
| Nov-08 | SH | NA | 30 | autumn | 1 | -0.32 | 13.84 |
| Dec-08 | SH | NA | 28 | autumn | 1 | -0.28 | 10.65 |
| Jan-09 | SH | NA | 31 | winter | 1 | -0.01 | 7.96 |
| Feb-09 | SH | NA | 28 | winter | 1 | 0.06 | 9.93 |
| Mar-09 | SH | NA | 31 | winter | 1 | 0.57 | 10.50 |
| Apr-09 | SH | NA | 30 | spring | 0 | -0.20 | 13.34 |
| May-09 | SH | NA | 31 | spring | 0 | 1.68 | 16.92 |
| Jun-09 | SH | 1.22 | 30 | spring | 1 | -1.21 | 19.68 |
| Jul-09 | SH | 0.50 | 31 | summer | 1 | -2.15 | 22.22 |
| Aug-09 | SH | 0.06 | 31 | summer | 1 | -0.19 | 22.06 |
| Sep-09 | SH | 0.60 | 30 | summer | 1 | 1.51 | 19.35 |
| Oct-09 | SH | 1.14 | 31 | autumn | 1 | -1.03 | 17.97 |
| Nov-09 | SH | 6.42 | 30 | autumn | 1 | -0.02 | 12.97 |
| Dec-09 | SH | 1.35 | 31 | autumn | 1 | -1.93 | 10.91 |
| Jan-10 | SH | 1.35 | 31 | winter | 1 | -1.11 | 9.35 |
| Feb-10 | SH | 2.50 | 28 | winter | 1 | -1.98 | 10.36 |
| Mar-10 | SH | 1.40 | 31 | winter | 1 | -0.88 | 12.46 |
| Apr-10 | SH | 0.98 | 30 | spring | 0 | -0.72 | 12.74 |
| May-10 | SH | 1.59 | 31 | spring | 0 | -1.49 | 15.90 |
| Jun-10 | SH | 2.54 | 30 | spring | 1 | -0.82 | 19.84 |
| Jul-10 | SH | 0.56 | 30 | summer | 1 | -0.42 | 21.07 |
| Aug-10 | SH | 0.05 | 31 | summer | 1 | -1.22 | 22.99 |
| Sep-10 | SH | 0.00 | 30 | summer | 1 | -0.79 | 20.89 |
| Oct-10 | SH | 0.02 | 29 | autumn | 1 | -0.93 | 18.94 |
| Nov-10 | SH | 4.94 | 28 | autumn | 1 | -1.62 | 16.29 |
| Dec-10 | SH | 3.23 | 31 | autumn | 1 | -1.85 | 13.06 |
| Jan-11 | SH | 1.38 | 31 | winter | 1 | -0.88 | 6.72 |
| Feb-11 | SH | 2.23 | 28 | winter | 1 | 0.70 | 7.79 |
| Mar-11 | SH | 3.07 | 31 | winter | 1 | 0.61 | 10.62 |
| Apr-11 | SH | 2.86 | 30 | spring | 0 | 2.48 | 12.53 |
| May-11 | SH | 1.49 | 31 | spring | 0 | -0.06 | 15.09 |
| Jun-11 | SH | 0.96 | 30 | spring | 1 | -1.28 | 19.02 |
| Jul-11 | SH | 0.61 | 31 | summer | 1 | -1.51 | 21.14 |
| Aug-11 | SH | 0.17 | 31 | summer | 1 | -1.35 | 21.64 |
| Sep-11 | SH | 0.53 | 30 | summer | 1 | 0.54 | 19.77 |
| Oct-11 | SH | 0.88 | 31 | autumn | 1 | 0.39 | 16.79 |
| Nov-11 | SH | 9.54 | 30 | autumn | 1 | 1.36 | 11.39 |
| Dec-11 | SH | 2.33 | 31 | autumn | 1 | 2.52 | 8.35 |
| Jan-12 | SH | NA | 30 | winter | 1 | 1.17 | 5.14 |
| Feb-12 | SH | NA | 28 | winter | 1 | 0.42 | 7.58 |
| Mar-12 | SH | NA | 31 | winter | 1 | 1.27 | 9.02 |
| Apr-12 | SH | NA | 30 | spring | 0 | 0.47 | 12.97 |
| May-12 | SH | NA | 31 | spring | 0 | -0.91 | 16.53 |
| Jun-12 | SH | NA | 30 | spring | 1 | -2.53 | 19.81 |
| Jul-12 | SH | NA | 31 | summer | 1 | -1.32 | 22.99 |
| Aug-12 | SH | NA | 31 | summer | 1 | -0.98 | 22.94 |
| Sep-12 | SH | NA | 30 | summer | 1 | -0.59 | 20.51 |
| Oct-12 | SH | NA | 27 | autumn | 1 | -2.06 | 18.83 |
| Nov-12 | SH | NA | 0 | autumn | 1 | -0.58 | NA |
| Jan-07 | Nablus | NA | 31 | winter | 1 | 0.22 | 3.98 |
| Feb-07 | Nablus | NA | 28 | winter | 1 | -0.47 | 6.60 |
| Mar-07 | Nablus | NA | 31 | winter | 1 | 1.44 | 8.90 |
| Apr-07 | Nablus | NA | 30 | spring | 0 | 0.17 | 12.25 |
| May-07 | Nablus | NA | 31 | spring | 0 | 0.66 | 16.37 |
| Jun-07 | Nablus | NA | 30 | spring | 1 | -1.31 | 19.77 |
| Jul-07 | Nablus | NA | 31 | summer | 1 | -0.58 | 20.89 |
| Aug-07 | Nablus | NA | 31 | summer | 1 | -0.14 | 21.29 |
| Sep-07 | Nablus | NA | 30 | summer | 1 | 0.72 | 19.43 |
| Oct-07 | Nablus | NA | 31 | autumn | 1 | 0.45 | 16.89 |
| Nov-07 | Nablus | NA | 29 | autumn | 1 | 0.58 | 12.55 |
| Dec-07 | Nablus | NA | 31 | autumn | 1 | 0.34 | 8.03 |
| Jan-08 | Nablus | NA | 30 | winter | 1 | 0.89 | 1.43 |
| Feb-08 | Nablus | NA | 29 | winter | 1 | 0.73 | 5.58 |
| Mar-08 | Nablus | NA | 31 | winter | 1 | 0.08 | 10.77 |
| Apr-08 | Nablus | NA | 30 | spring | 0 | -1.07 | 13.42 |
| May-08 | Nablus | NA | 31 | spring | 0 | -1.73 | 15.69 |
| Jun-08 | Nablus | NA | 30 | spring | 1 | -1.39 | 19.15 |
| Jul-08 | Nablus | NA | 31 | summer | 1 | -1.27 | 21.33 |
| Aug-08 | Nablus | NA | 31 | summer | 1 | -1.16 | 20.52 |
| Sep-08 | Nablus | NA | 30 | summer | 1 | 1.02 | 18.79 |
| Oct-08 | Nablus | NA | 31 | autumn | 1 | -0.04 | 15.38 |
| Nov-08 | Nablus | NA | 30 | autumn | 1 | -0.32 | 11.76 |
| Dec-08 | Nablus | NA | 28 | autumn | 1 | -0.28 | 7.77 |
| Jan-09 | Nablus | NA | 31 | winter | 1 | -0.01 | 5.29 |
| Feb-09 | Nablus | NA | 28 | winter | 1 | 0.06 | 7.75 |
| Mar-09 | Nablus | NA | 31 | winter | 1 | 0.57 | 9.37 |
| Apr-09 | Nablus | NA | 30 | spring | 0 | -0.20 | 11.20 |
| May-09 | Nablus | NA | 31 | spring | 0 | 1.68 | 16.32 |
| Jun-09 | Nablus | NA | 30 | spring | 1 | -1.21 | 19.25 |
| Jul-09 | Nablus | NA | 31 | summer | 1 | -2.15 | 21.70 |
| Aug-09 | Nablus | NA | 31 | summer | 1 | -0.19 | 21.47 |
| Sep-09 | Nablus | NA | 30 | summer | 1 | 1.51 | 18.63 |
| Oct-09 | Nablus | NA | 31 | autumn | 1 | -1.03 | 16.85 |
| Nov-09 | Nablus | NA | 30 | autumn | 1 | -0.02 | 11.80 |
| Dec-09 | Nablus | NA | 31 | autumn | 1 | -1.93 | 9.41 |
| Jan-10 | Nablus | NA | 31 | winter | 1 | -1.11 | 5.81 |
| Feb-10 | Nablus | 0.03 | 28 | winter | 1 | -1.98 | 7.77 |
| Mar-10 | Nablus | 0.03 | 31 | winter | 1 | -0.88 | 10.28 |
| Apr-10 | Nablus | 0.09 | 30 | spring | 0 | -0.72 | 12.95 |
| May-10 | Nablus | 0.15 | 31 | spring | 0 | -1.49 | 15.64 |
| Jun-10 | Nablus | 0.28 | 30 | spring | 1 | -0.82 | 19.67 |
| Jul-10 | Nablus | 0.49 | 30 | summer | 1 | -0.42 | 20.48 |
| Aug-10 | Nablus | 7.27 | 31 | summer | 1 | -1.22 | 22.91 |
| Sep-10 | Nablus | 0.63 | 30 | summer | 1 | -0.79 | 20.36 |
| Oct-10 | Nablus | 1.86 | 29 | autumn | 1 | -0.93 | 18.03 |
| Nov-10 | Nablus | 6.01 | 28 | autumn | 1 | -1.62 | 13.94 |
| Dec-10 | Nablus | 0.63 | 31 | autumn | 1 | -1.85 | 9.93 |
| Jan-11 | Nablus | 0.07 | 31 | winter | 1 | -0.88 | 3.80 |
| Feb-11 | Nablus | 0.01 | 28 | winter | 1 | 0.70 | 6.12 |
| Mar-11 | Nablus | 0.00 | 31 | winter | 1 | 0.61 | 9.28 |
| Apr-11 | Nablus | 0.00 | 30 | spring | 0 | 2.48 | 11.72 |
| May-11 | Nablus | 0.00 | 31 | spring | 0 | -0.06 | 14.36 |
| Jun-11 | Nablus | 0.02 | 30 | spring | 1 | -1.28 | 18.73 |
| Jul-11 | Nablus | 1.91 | 31 | summer | 1 | -1.51 | 21.04 |
| Aug-11 | Nablus | 2.61 | 31 | summer | 1 | -1.35 | 21.47 |
| Sep-11 | Nablus | 0.65 | 30 | summer | 1 | 0.54 | 19.37 |
| Oct-11 | Nablus | 1.75 | 31 | autumn | 1 | 0.39 | 15.66 |
| Nov-11 | Nablus | 1.33 | 30 | autumn | 1 | 1.36 | 9.25 |
| Dec-11 | Nablus | 0.06 | 31 | autumn | 1 | 2.52 | 6.43 |
| Jan-12 | Nablus | 0.07 | 30 | winter | 1 | 1.17 | 3.75 |
| Feb-12 | Nablus | 0.03 | 28 | winter | 1 | 0.42 | 5.80 |
| Mar-12 | Nablus | 0.04 | 31 | winter | 1 | 1.27 | 8.63 |
| Apr-12 | Nablus | 0.02 | 30 | spring | 0 | 0.47 | 12.62 |
| May-12 | Nablus | 0.00 | 31 | spring | 0 | -0.91 | 16.15 |
| Jun-12 | Nablus | 0.00 | 30 | spring | 1 | -2.53 | 19.60 |
| Jul-12 | Nablus | 0.65 | 31 | summer | 1 | -1.32 | 22.80 |
| Aug-12 | Nablus | 0.41 | 31 | summer | 1 | -0.98 | 22.47 |
| Sep-12 | Nablus | 0.18 | 30 | summer | 1 | -0.59 | 19.99 |
| Oct-12 | Nablus | 1.71 | 27 | autumn | 1 | -2.06 | 18.20 |
| Nov-12 | Nablus | 3.24 | 0 | autumn | 1 | -0.58 | NA |
| Jan-07 | Tubas | NA | 31 | winter | 1 | 0.22 | 3.19 |
| Feb-07 | Tubas | NA | 28 | winter | 1 | -0.47 | 5.92 |
| Mar-07 | Tubas | NA | 31 | winter | 1 | 1.44 | 8.42 |
| Apr-07 | Tubas | NA | 30 | spring | 0 | 0.17 | 11.86 |
| May-07 | Tubas | NA | 31 | spring | 0 | 0.66 | 16.20 |
| Jun-07 | Tubas | NA | 30 | spring | 1 | -1.31 | 19.40 |
| Jul-07 | Tubas | NA | 31 | summer | 1 | -0.58 | 21.26 |
| Aug-07 | Tubas | NA | 31 | summer | 1 | -0.14 | 22.03 |
| Sep-07 | Tubas | NA | 30 | summer | 1 | 0.72 | 19.96 |
| Oct-07 | Tubas | NA | 31 | autumn | 1 | 0.45 | 17.30 |
| Nov-07 | Tubas | NA | 29 | autumn | 1 | 0.58 | 12.29 |
| Dec-07 | Tubas | NA | 31 | autumn | 1 | 0.34 | 7.66 |
| Jan-08 | Tubas | NA | 30 | winter | 1 | 0.89 | 1.01 |
| Feb-08 | Tubas | NA | 29 | winter | 1 | 0.73 | 5.57 |
| Mar-08 | Tubas | NA | 31 | winter | 1 | 0.08 | 10.71 |
| Apr-08 | Tubas | NA | 30 | spring | 0 | -1.07 | 12.66 |
| May-08 | Tubas | NA | 31 | spring | 0 | -1.73 | 16.12 |
| Jun-08 | Tubas | NA | 30 | spring | 1 | -1.39 | 19.74 |
| Jul-08 | Tubas | NA | 31 | summer | 1 | -1.27 | 21.89 |
| Aug-08 | Tubas | NA | 31 | summer | 1 | -1.16 | 21.27 |
| Sep-08 | Tubas | NA | 30 | summer | 1 | 1.02 | 19.27 |
| Oct-08 | Tubas | NA | 31 | autumn | 1 | -0.04 | 15.65 |
| Nov-08 | Tubas | NA | 30 | autumn | 1 | -0.32 | 11.34 |
| Dec-08 | Tubas | NA | 28 | autumn | 1 | -0.28 | 7.14 |
| Jan-09 | Tubas | NA | 31 | winter | 1 | -0.01 | 5.05 |
| Feb-09 | Tubas | NA | 28 | winter | 1 | 0.06 | 6.84 |
| Mar-09 | Tubas | NA | 31 | winter | 1 | 0.57 | 8.60 |
| Apr-09 | Tubas | NA | 30 | spring | 0 | -0.20 | 11.55 |
| May-09 | Tubas | NA | 31 | spring | 0 | 1.68 | 16.06 |
| Jun-09 | Tubas | NA | 30 | spring | 1 | -1.21 | 19.85 |
| Jul-09 | Tubas | NA | 31 | summer | 1 | -2.15 | 22.48 |
| Aug-09 | Tubas | NA | 31 | summer | 1 | -0.19 | 22.36 |
| Sep-09 | Tubas | 0.35 | 30 | summer | 1 | 1.51 | 19.14 |
| Oct-09 | Tubas | 1.02 | 31 | autumn | 1 | -1.03 | 17.18 |
| Nov-09 | Tubas | 0.82 | 30 | autumn | 1 | -0.02 | 11.61 |
| Dec-09 | Tubas | 0.71 | 31 | autumn | 1 | -1.93 | 9.17 |
| Jan-10 | Tubas | 0.61 | 31 | winter | 1 | -1.11 | 5.81 |
| Feb-10 | Tubas | 1.31 | 28 | winter | 1 | -1.98 | 7.73 |
| Mar-10 | Tubas | 0.38 | 31 | winter | 1 | -0.88 | 10.10 |
| Apr-10 | Tubas | 0.28 | 30 | spring | 0 | -0.72 | 13.14 |
| May-10 | Tubas | 0.88 | 31 | spring | 0 | -1.49 | 15.42 |
| Jun-10 | Tubas | 1.70 | 30 | spring | 1 | -0.82 | 20.09 |
| Jul-10 | Tubas | 0.20 | 30 | summer | 1 | -0.42 | 21.22 |
| Aug-10 | Tubas | 0.11 | 31 | summer | 1 | -1.22 | 23.64 |
| Sep-10 | Tubas | 0.00 | 30 | summer | 1 | -0.79 | 21.25 |
| Oct-10 | Tubas | 0.01 | 29 | autumn | 1 | -0.93 | 18.13 |
| Nov-10 | Tubas | 0.03 | 28 | autumn | 1 | -1.62 | 13.80 |
| Dec-10 | Tubas | 2.21 | 31 | autumn | 1 | -1.85 | 9.65 |
| Jan-11 | Tubas | 1.34 | 31 | winter | 1 | -0.88 | 3.57 |
| Feb-11 | Tubas | 1.99 | 28 | winter | 1 | 0.70 | 5.99 |
| Mar-11 | Tubas | 3.33 | 31 | winter | 1 | 0.61 | 9.07 |
| Apr-11 | Tubas | 1.28 | 30 | spring | 0 | 2.48 | 10.87 |
| May-11 | Tubas | 0.71 | 31 | spring | 0 | -0.06 | 14.82 |
| Jun-11 | Tubas | 0.58 | 30 | spring | 1 | -1.28 | 19.17 |
| Jul-11 | Tubas | 4.26 | 31 | summer | 1 | -1.51 | 21.88 |
| Aug-11 | Tubas | 0.48 | 31 | summer | 1 | -1.35 | 22.35 |
| Sep-11 | Tubas | 0.13 | 30 | summer | 1 | 0.54 | 20.08 |
| Oct-11 | Tubas | 0.05 | 31 | autumn | 1 | 0.39 | 15.79 |
| Nov-11 | Tubas | 4.55 | 30 | autumn | 1 | 1.36 | 8.76 |
| Dec-11 | Tubas | 6.00 | 31 | autumn | 1 | 2.52 | 5.82 |
| Jan-12 | Tubas | 0.97 | 30 | winter | 1 | 1.17 | 3.15 |
| Feb-12 | Tubas | 1.93 | 28 | winter | 1 | 0.42 | 5.22 |
| Mar-12 | Tubas | 2.55 | 31 | winter | 1 | 1.27 | 8.10 |
| Apr-12 | Tubas | 2.97 | 30 | spring | 0 | 0.47 | 12.72 |
| May-12 | Tubas | 1.50 | 31 | spring | 0 | -0.91 | 15.96 |
| Jun-12 | Tubas | 1.27 | 30 | spring | 1 | -2.53 | 20.00 |
| Jul-12 | Tubas | 10.00 | 31 | summer | 1 | -1.32 | 23.59 |
| Aug-12 | Tubas | 5.61 | 31 | summer | 1 | -0.98 | 23.39 |
| Sep-12 | Tubas | 1.21 | 30 | summer | 1 | -0.59 | 20.60 |
| Oct-12 | Tubas | 2.68 | 27 | autumn | 1 | -2.06 | 18.75 |
| Nov-12 | Tubas | 4.16 | 0 | autumn | 1 | -0.58 | NA |
| Jan-07 | Tulkarem | NA | 31 | winter | 1 | 0.22 | 6.40 |
| Feb-07 | Tulkarem | NA | 28 | winter | 1 | -0.47 | 8.86 |
| Mar-07 | Tulkarem | NA | 31 | winter | 1 | 1.44 | 11.22 |
| Apr-07 | Tulkarem | NA | 30 | spring | 0 | 0.17 | 14.10 |
| May-07 | Tulkarem | NA | 31 | spring | 0 | 0.66 | 18.37 |
| Jun-07 | Tulkarem | NA | 30 | spring | 1 | -1.31 | 21.22 |
| Jul-07 | Tulkarem | NA | 31 | summer | 1 | -0.58 | 23.47 |
| Aug-07 | Tulkarem | NA | 31 | summer | 1 | -0.14 | 24.09 |
| Sep-07 | Tulkarem | NA | 30 | summer | 1 | 0.72 | 21.84 |
| Oct-07 | Tulkarem | NA | 31 | autumn | 1 | 0.45 | 18.93 |
| Nov-07 | Tulkarem | NA | 29 | autumn | 1 | 0.58 | 14.66 |
| Dec-07 | Tulkarem | NA | 31 | autumn | 1 | 0.34 | 10.89 |
| Jan-08 | Tulkarem | NA | 30 | winter | 1 | 0.89 | 4.39 |
| Feb-08 | Tulkarem | NA | 29 | winter | 1 | 0.73 | 8.71 |
| Mar-08 | Tulkarem | NA | 31 | winter | 1 | 0.08 | 12.48 |
| Apr-08 | Tulkarem | NA | 30 | spring | 0 | -1.07 | 15.26 |
| May-08 | Tulkarem | NA | 31 | spring | 0 | -1.73 | 17.53 |
| Jun-08 | Tulkarem | NA | 30 | spring | 1 | -1.39 | 21.45 |
| Jul-08 | Tulkarem | NA | 31 | summer | 1 | -1.27 | 23.93 |
| Aug-08 | Tulkarem | NA | 31 | summer | 1 | -1.16 | 23.13 |
| Sep-08 | Tulkarem | NA | 30 | summer | 1 | 1.02 | 21.21 |
| Oct-08 | Tulkarem | NA | 31 | autumn | 1 | -0.04 | 17.70 |
| Nov-08 | Tulkarem | NA | 30 | autumn | 1 | -0.32 | 14.61 |
| Dec-08 | Tulkarem | NA | 28 | autumn | 1 | -0.28 | 10.99 |
| Jan-09 | Tulkarem | NA | 31 | winter | 1 | -0.01 | 6.13 |
| Feb-09 | Tulkarem | NA | 28 | winter | 1 | 0.06 | 9.19 |
| Mar-09 | Tulkarem | NA | 31 | winter | 1 | 0.57 | 11.11 |
| Apr-09 | Tulkarem | NA | 30 | spring | 0 | -0.20 | 13.70 |
| May-09 | Tulkarem | NA | 31 | spring | 0 | 1.68 | 18.14 |
| Jun-09 | Tulkarem | NA | 30 | spring | 1 | -1.21 | 21.64 |
| Jul-09 | Tulkarem | NA | 31 | summer | 1 | -2.15 | 24.27 |
| Aug-09 | Tulkarem | NA | 31 | summer | 1 | -0.19 | 24.43 |
| Sep-09 | Tulkarem | NA | 30 | summer | 1 | 1.51 | 21.13 |
| Oct-09 | Tulkarem | NA | 31 | autumn | 1 | -1.03 | 19.25 |
| Nov-09 | Tulkarem | NA | 30 | autumn | 1 | -0.02 | 14.53 |
| Dec-09 | Tulkarem | NA | 31 | autumn | 1 | -1.93 | 12.07 |
| Jan-10 | Tulkarem | NA | 31 | winter | 1 | -1.11 | 8.62 |
| Feb-10 | Tulkarem | 0.15 | 28 | winter | 1 | -1.98 | 10.63 |
| Mar-10 | Tulkarem | 0.14 | 31 | winter | 1 | -0.88 | 13.12 |
| Apr-10 | Tulkarem | 0.38 | 30 | spring | 0 | -0.72 | 14.64 |
| May-10 | Tulkarem | 0.52 | 31 | spring | 0 | -1.49 | 17.67 |
| Jun-10 | Tulkarem | 0.88 | 30 | spring | 1 | -0.82 | 21.21 |
| Jul-10 | Tulkarem | 0.44 | 30 | summer | 1 | -0.42 | 23.36 |
| Aug-10 | Tulkarem | 0.37 | 31 | summer | 1 | -1.22 | 25.20 |
| Sep-10 | Tulkarem | 0.02 | 30 | summer | 1 | -0.79 | 22.90 |
| Oct-10 | Tulkarem | 0.03 | 29 | autumn | 1 | -0.93 | 20.19 |
| Nov-10 | Tulkarem | 0.52 | 28 | autumn | 1 | -1.62 | 16.33 |
| Dec-10 | Tulkarem | 2.21 | 31 | autumn | 1 | -1.85 | 12.40 |
| Jan-11 | Tulkarem | 1.34 | 31 | winter | 1 | -0.88 | 7.50 |
| Feb-11 | Tulkarem | 0.54 | 28 | winter | 1 | 0.70 | 8.76 |
| Mar-11 | Tulkarem | 0.75 | 31 | winter | 1 | 0.61 | 11.60 |
| Apr-11 | Tulkarem | 0.67 | 30 | spring | 0 | 2.48 | 13.83 |
| May-11 | Tulkarem | 0.10 | 31 | spring | 0 | -0.06 | 16.91 |
| Jun-11 | Tulkarem | 0.29 | 30 | spring | 1 | -1.28 | 20.65 |
| Jul-11 | Tulkarem | 1.88 | 31 | summer | 1 | -1.51 | 23.59 |
| Aug-11 | Tulkarem | 1.88 | 31 | summer | 1 | -1.35 | 24.24 |
| Sep-11 | Tulkarem | 0.49 | 30 | summer | 1 | 0.54 | 21.91 |
| Oct-11 | Tulkarem | 0.43 | 31 | autumn | 1 | 0.39 | 18.18 |
| Nov-11 | Tulkarem | 4.67 | 30 | autumn | 1 | 1.36 | 11.68 |
| Dec-11 | Tulkarem | 2.86 | 31 | autumn | 1 | 2.52 | 9.52 |
| Jan-12 | Tulkarem | NA | 30 | winter | 1 | 1.17 | 4.83 |
| Feb-12 | Tulkarem | NA | 28 | winter | 1 | 0.42 | 7.98 |
| Mar-12 | Tulkarem | NA | 31 | winter | 1 | 1.27 | 9.58 |
| Apr-12 | Tulkarem | NA | 30 | spring | 0 | 0.47 | 14.06 |
| May-12 | Tulkarem | NA | 31 | spring | 0 | -0.91 | 18.12 |
| Jun-12 | Tulkarem | NA | 30 | spring | 1 | -2.53 | 21.65 |
| Jul-12 | Tulkarem | NA | 31 | summer | 1 | -1.32 | 25.18 |
| Aug-12 | Tulkarem | NA | 31 | summer | 1 | -0.98 | 25.34 |
| Sep-12 | Tulkarem | NA | 30 | summer | 1 | -0.59 | 22.71 |
| Oct-12 | Tulkarem | NA | 27 | autumn | 1 | -2.06 | 20.96 |
| Nov-12 | Tulkarem | NA | 0 | autumn | 1 | -0.58 | NA |
