# Supplementary material for: Olive Fruit Fly (Bactrocera oleae) Population Dynamics in the Eastern Mediterranean: Influence of Exogenous Uncertainty on a Monophagous Frugivorous Insect
Source: PLoS One. 2015 May 26;10(5):e0127798. doi: 10.1371/journal.pone.0127798 (PMC4444365; doi:10.1371/journal.pone.0127798)
Supplement: S2 Table — Linear trend analysis for monthly average night land surface temperatures between February 2007 and November 2012 at five locations in the Eastern Mediterranean region. The linear trend analyses indicated that there were no temporal trends in the monthly North Atlantic Oscillation index (adjusted R 2 = -0.01468, t = -0.043, p = 0.965) and the monthly-average night land surface temperatures. For data sources, see Methods. (DOC) [file pone.0127798.s004.doc]

**S2 Table**

Title

*Olive fruit fly (Bactrocera oleae) population dynamics in the Eastern Mediterranean: Influence of exogenous uncertainty on a monophagous frugivorous insect*

Authors

Mariano Ordano, Izhar Engelhard, Polychronis Rempoulakis, Esther Nemny-Lavy, Moshe Blum, Sami Yasin, Itamar Lensky, Nikos T. Papadopoulos, David Nestel*

*Corresponding Author

Temporal explorations and trend analysis applied to climatic time-series. For data sources, see Materials and Methods.

Summary of the linear trend analyses that indicated that there were no temporal trends for the monthly North Atlantic Oscillation index (adjusted *R^2^* = -0.01468, *t* = -0.043, *p* = 0.965), and on the monthly average night land surface temperature (monthly averaged between February 2007 and November 2012). Results below show the estimates for the five locations in the Eastern Mediterranean region.

| Site | Adjusted *R*^2^ | Estimate | SE | *t* | *p* |
| --- | --- | --- | --- | --- | --- |
| Lahav | 0.001 | -0.025 | 0.025 | -1.032 | 0.307 |
| Sha'ar HaGai | -0.030 | -0.025 | 0.061 | -0.405 | 0.689 |
| Nablus | -0.030 | -0.019 | 0.063 | -0.297 | 0.769 |
| Tubas | -0.012 | 0.041 | 0.053 | 0.769 | 0.447 |
| Tulkarem | 0.079 | -0.174 | 0.104 | -1.676 | 0.109 |
