# Supplementary material for: Olive Fruit Fly (Bactrocera oleae) Population Dynamics in the Eastern Mediterranean: Influence of Exogenous Uncertainty on a Monophagous Frugivorous Insect
Source: PLoS One. 2015 May 26;10(5):e0127798. doi: 10.1371/journal.pone.0127798 (PMC4444365; doi:10.1371/journal.pone.0127798)
Supplement: S3 Table — Models carried out to examine the influence of denso-dependence, climatic (local and global) factors, fruit occurrence and seasonality on the rate of change in Bactrocera oleae populations in five locations in the Eastern Mediterranean region. Description of the procedure used to select the optimal model. Tables of competing models for each site are showed. Selected models are marked with an asterisk. Main basic model (Eq 2 of Methods): Rt = log(MFTt-1) + nLST + NAOi + fruit. BIC: Bayesian Information Criteria. ARMA: order of the autoregressive moving average (p, q). The variance structure follows the R code used for generalized-least-squares models. (DOC) [file pone.0127798.s005.doc]

**S3 Table**

Title

*Olive fruit fly (Bactrocera oleae) population dynamics in the Eastern Mediterranean: Influence of exogenous uncertainty on a monophagous frugivorous insect*

Authors

Mariano Ordano, Izhar Engelhard, Polychronis Rempoulakis, Esther Nemny-Lavy, Moshe Blum, Sami Yasin, Itamar Lensky, Nikos T. Papadopoulos, David Nestel*

*Corresponding Author

Short description of the how we selected the optimal generalized-least-squares model to describe the influence of denso-dependence, climatic (local and global) factors, the presence or absence of fruit, and seasonality on the rate of change in *Bactrocera oleae* populations in five locations of the Eastern Mediterranean region. See Materials and Methods for details.

**Tables of competing models for each site**

Selected models are marked with an asterisk.

Main basic model (Equation [2] from the Materials and Methods):

*R_t_ = log(MFT_t-1_) + nLST + NAOi + fruit*

BIC: Bayesian Information Criteria. ARMA: order of the autoregressive moving average (p, q). Variance structure follows R code used in generalized least square models.

**Description of the procedure to select the optimal model**

(1) Fit generalized-least-squares models by ML. [R function: *gls*].

(2) Select the best model based on the lower BIC value and significant differences indicated by the ML test. If two models do not differ, select the most parsimonious one (for example, an ARMA structure (1, 0) when it was no different from a (1,1) alternative ARMA structure). [R function: *anova*].

(3) Fit the selected generalized least squares model by REML. [R function: *gls*].

(4) Use the Shapiro-Wilk test to validate the estimation run in (3). Check white noise, Pearson residuals and variance structures. [R functions: *shapiro.test*, *plot*, *ACF*].

(5) If the validation does not reveal any Gaussian errors, return to Step 1 and fit models with alternative structures, and repeat the process. If the validation reveals Gaussian errors, but also variance effects by a given variable, return to Step 1 and fit models with alternative structures (in this case with weights related to the variable which variance effects were observed), and repeat the process. If diagnostics indicate both Gaussian errors and negligible variance effects (i.e., shared standard deviation between levels), you have identified the optimal model.

**Lahav**

| ID model | BIC | ARMA | Variance structure |
| --- | --- | --- | --- |
| g7 * | 146.746 | (2,2) | varComb(varIdent(form=~1|season), varIdent(form=~1|year), varFixed(~nLST)) |
| g13 | 148.810 | (2,2) | varFixed(~nLST) |
| g6 | 152.213 | (2,2) | Null |
| g8 | 156.134 | (2,2) | varComb(varIdent(form=~1|season), varIdent(form=~1|year)) |
| g12 | 159.555 | (2,2) | varIdent(form=~1|season) |
| g9 | 163.623 | (2,2) | varComb(varIdent(form=~1|season), varFixed(~nLST)) |
| g1 | 171.688 | (1,0) | Null |
| g3 | 174.721 | (1,1) | Null |
| g2 | 174.804 | (2,0) | Null |
| g4 | 175.259 | (1,2) | Null |
| g5 | 178.746 | (2,1) | Null |
| g10 | 182.280 | (2,2) | varComb(varIdent(form=~1|year), varFixed(~nLST)) |

**Sha’ar HaGai**

| ID model | BIC | ARMA | Variance structure |
| --- | --- | --- | --- |
| g6 | 96.261 | (2,2) | Null |
| g9 *‡ | 96.798 | (2,2) | varComb(varIdent(form=~1|season), varFixed(~nLST)) |
| g12 | 97.432 | (2,2) | varIdent(form=~1|season) |
| g7 | 102.325 | (2,2) | varComb(varIdent(form=~1|season), varIdent(form=~1|year), varFixed(~nLST)) |
| g5 | 102.394 | (2,1) | Null |
| g2 | 102.677 | (2,0) | Null |
| g8 | 103.538 | (2,2) | varComb(varIdent(form=~1|season), varIdent(form=~1|year)) |
| g13 | 106.356 | (2,2) | varFixed(~nLST) |
| g1 | 106.841 | (1,0) | Null |
| g11 | 108.417 | (2,2) | varIdent(form=~1|season) |
| g3 | 108.940 | (1,1) | Null |
| g10 | 110.985 | (2,2) | varComb(varIdent(form=~1|year), varFixed(~nLST)) |

‡ Model g6 had non-normal residuals. We chose Model g9.

**Nablus**

| ID model | BIC | ARMA | Variance structure |
| --- | --- | --- | --- |
| g13 * | 104.720 | (2,0) | varFixed(~nLST) |
| g2 | 105.575 | (2,0) | Null |
| g5 | 108.730 | (2,1) | Null |
| g10 | 109.094 | (2,0) | varComb(varIdent(form=~1|year), varFixed(~nLST)) |
| g3 | 109.687 | (1,1) | Null |
| g11 | 111.245 | (2,0) | varIdent(form=~1|season) |
| g6 | 111.896 | (2,2) | Null |
| g1 | 112.386 | (1,0) | Null |
| g12 | 112.574 | (2,0) | varIdent(form=~1|season) |
| g8 | 114.191 | (2,0) | varComb(varIdent(form=~1|season), varIdent(form=~1|year)) |
| g9 | 114.465 | (2,0) | varComb(varIdent(form=~1|season), varFixed(~nLST)) |
| g7 | 117.042 | (2,0) | varComb(varIdent(form=~1|season), varIdent(form=~1|year), varFixed(~nLST)) |

**Tubas**

| ID model | BIC | ARMA | Variance structure |
| --- | --- | --- | --- |
| g9 * | 133.959 | (1,0) | varComb(varIdent(form=~1|season), varFixed(~nLST)) |
| g12 | 134.578 | (1,0) | varIdent(form=~1|season) |
| g13 | 135.710 | (1,0) | varFixed(~nLST) |
| g1 | 138.746 | (1,0) | Null |
| g2 | 140.954 | (2,0) | Null |
| g5 | 141.340 | (2,1) | Null |
| g3 | 142.168 | (1,1) | Null |
| g6 | 144.451 | (2,2) | Null |

**Tulkarem**

| ID model | BIC | ARMA | Variance structure |
| --- | --- | --- | --- |
| g13 * | 75.512 | (2,0) | varFixed(~nLST) |
| g2 | 76.658 | (2,0) | Null |
| g16 | 77.820 | (2,0) | varComb(varIdent(form=~1|year), varFixed(~NAOI)) |
| g11 | 78.259 | (2,0) | varIdent(form=~1|season) |
| g10 | 78.388 | (2,0) | varComb(varIdent(form=~1|year), varFixed(~nLST)) |
| g17 | 78.635 | (2,0) | varFixed(~NAOI) |
| g5 | 79.507 | (2,1) | Null |
| g1 | 79.765 | (1,0) | Null |
| g3 | 80.451 | (1,1) | Null |
| g6 | 80.517 | (2,2) | Null |
| g9 | 82.389 | (2,0) | varComb(varIdent(form=~1|season), varFixed(~nLST)) |
| g12 | 82.558 | (2,0) | varIdent(form=~1|season) |
| g14 | 83.902 | (2,0) | varComb(varIdent(form=~1|season), varIdent(form=~1|year), varFixed(~NAOI)) |
| g8 | 84.012 | (2,0) | varComb(varIdent(form=~1|season), varIdent(form=~1|year)) |
| g7 | 85.472 | (2,0) | varComb(varIdent(form=~1|season), varIdent(form=~1|year), varFixed(~nLST)) |
| g15 | 85.489 | (2,0) | varComb(varIdent(form=~1|season), varFixed(~NAOI)) |
